# The mood stabilizers lithium and valproate disrupt hepatic and intestinal farnesoid X receptor signalling and increase bile synthesis in the rat

**DOI:** 10.1113/EP092451

**Published:** 2025-03-28

**Authors:** Sofia Cussotto, Anna V. Golubeva, Alvaro Lopez Gallardo, Thomaz F. S. Bastiaanssen, Alexander V. Zhdanov, Gerard M. Moloney, Caitriona Scaife, Jane A. English, Susan A. Joyce, Timothy G. Dinan, John F. Cryan

**Affiliations:** ^1^ APC Microbiome Ireland University College Cork Cork Ireland; ^2^ Department of Anatomy and Neuroscience University College Cork Cork Ireland; ^3^ School of Biochemistry and Cell Biology University College Cork Cork Ireland; ^4^ School of Pharmacy University College Cork Cork Ireland; ^5^ Mass Spectrometry Core UCD Conway Institute Dublin Ireland; ^6^ INFANT research centre CUH Cork Ireland; ^7^ Department of Psychiatry and Neurobehavioural Science University College Cork Cork Ireland

**Keywords:** ASBT, bile acid diarrhoea, bile acids, CYP7A1, FGF19, FXR, lithium, liver proteome, mood stabilizer, valproate

## Abstract

The mood stabilizers lithium and valproate are psychotropic medications widely used in clinical practice. Despite their proven benefits, many individuals stop their treatment due to the adverse effects. Chronic diarrhoea is a common reason for discontinuation of these drugs; however, the underlying mechanisms are unknown. Excessive loss of bile acids (BA) into the colon is a major cause of diarrhoea. Therefore, we aimed to investigate the effects of these drugs on BA metabolism. We measured BA levels in the liver, plasma and faeces of Sprague–Dawley rats treated with lithium or valproate for 4 weeks. Next, we analysed changes in the expression of genes and proteins involved in BA production and enterohepatic circulation. Lithium and valproate markedly increased BA levels across all body sites. This was accompanied by the up‐regulation of hepatic cytochrome P450 7A1 (Cyp7a1), the rate‐limiting enzyme in *de novo* BA synthesis. Under normal conditions, elevated levels of BAs suppress Cyp7a1 via activation of the hepatic farnesoid X receptor (Fxr)/small heterodimer partner (Shp) and intestinal Fxr/fibroblast growth factor 19 (Fgf19) pathways. This signalling was disrupted in both treatment groups. The Fxr‐mediated responses in the expression of Ntcp, Asbt, Ilbp and Ostα/β bile transporters were also affected by treatment. In conclusion, lithium and valproate disrupted farnesoid X receptor signalling at the hepatic and intestinal levels, inducing sustained overproduction of bile in rats. These findings provide novel insights into the peripheral effects of these drugs. Given that similar changes in bile circuits underlie the pathophysiology of primary BA diarrhoea in humans, this study suggests a potential mechanism behind chronic diarrhoea in patients undergoing lithium or valproate therapy.

## Abstract (highlights)

- **What is the central question of this study?**
  How does chronic treatment with the mood stabilizers lithium or valproate affect bile acid metabolism and farnesoid X receptor (FXR) signalling in rats?
- **What is the main finding and its importance?**
  Lithium and valproate disrupted FXR signalling at both hepatic and intestinal levels, leading to increased *de novo* bile synthesis and a larger bile pool. Given the importance of bile acids in regulating gut physiology, these findings suggest a potential mechanism behind gastrointestinal distress, such as diarrhoea, in patients undergoing lithium or valproate therapy.

## 1 INTRODUCTION

Lithium and valproate are two common psychotropic drugs used as mood stabilizers and, in the case of valproate, as antiepileptics. Despite many years of research, the mechanisms of action of these drugs remain largely unknown, partly due to the unique nature of each drug. Lithium is a simple element that does not bind to any specific receptor. Instead, it modifies intracellular signalling by competing with Na^+^ and Mg^2+^ and inhibiting a series of metalloproteins such as G‐proteins, inositol phosphatases, adenylate cyclase, phospholipase C, protein kinases and so forth (Jakobsson et al., 2017; Pasquali et al., 2010). Valproate, a branched short‐chain fatty acid, can target extracellular signal‐regulated kinase, protein kinase C and glycogen synthase kinase‐3 pathways, as well as histone deacetylases and Na^+^‐dependent channels (Rosenberg, 2007; Tomson et al., 2016).

Due to their non‐specific mechanisms of action, both drugs display a broad range of central and peripheral side effects, many of which are still poorly understood. One well‐recognised side effect of both drugs is diarrhoea (Gitlin, 2016; Jahromi et al., 2011), which in one study represented the third most common reason for discontinuing lithium therapy (Öhlund et al., 2018). Although less evidence is available for valproate, discontinuation data from clinical studies support its gut‐targeted side effects (Jahromi et al., 2011; Mazurkiewicz‐Bełdzińska et al., 2010). Chronic diarrhoea, often categorized as bile acid diarrhoea (BAD) (Walters & Pattni, 2010), can result from excessive loss of bile acids (BA) in the colon, leading to fluid secretion, increased colonic motility and mucosal permeability (Farrugia & Arasaradnam, 2021; Hegyi et al., 2018; Oduyebo & Camilleri, 2017). Additionally, both lithium and valproate have been associated with body weight gain of indefinite pathogenesis (Ackerman & Nolan, 1998), while BAs play crucial roles in regulating lipid and glucose metabolism (Lefebvre et al., 2009).

Furthermore, we have previously shown that chronic administration of lithium and valproate in rats induced significant changes in the composition of the gut microbiota (Cussotto et al., 2019). In another study, the antidepressant drug paroxetine affected microbiota α‐diversity along with faecal bile acid levels in mice (Dethloff et al., 2020). Gut bacteria play a key role in BA metabolism, transforming primary BAs into secondary and tertiary bile moieties (Long et al., 2017). These transformations can shape the signalling fingerprint of BA by shifting their capacity to activate farnesoid X (FXR) receptor and Takeda G protein‐coupled (TGR5) receptor in the gut (Perino et al., 2021). Overall, this body of knowledge inspired us to investigate the effects of lithium and valproate on the synthesis and enterohepatic circulation of bile.

Considering the spectrum of their activity, both lithium and valproate have the potential to impact bile metabolism at different levels, including Na^+^‐dependent transporters (apical sodium–bile acid transporter (ASBT), Na^+^–taurocholate cotransporting polypeptide (NTCP)), ATP‐dependent transporters (bile salt export pump (BSEP), multidrug resistance‐associated protein 2 (MRP2)) and epigenetic mechanisms (FXR) (Kemper, 2011; Rosenberg, 2007; Tomson et al., 2016). However, our understanding of the effects of psychotropic drugs on bile physiology is limited. One study in rats showed a choleretic effect of valproate following an acute intravenous injection (Watkins & Klaassen, 1981). In line with this, valproate has been shown to induce some degree of hepatotoxicity both in humans and in rodents (Eadie et al., 1988; Fu et al., 2019; Greenberg, 2003). Another study has shown that lithium‐induced cation loss occurs not only via urine and faeces but also via bile (Kersten & Barth, 1985).

To address this gap, we used biological samples from our previous study on chronic administration of lithium and valproate in rats (Cussotto et al., 2019). We aimed to (1) investigate the effects of these drugs on the BA pool in the liver, plasma and faeces and (2) examine the mechanisms of drug‐induced effects in the liver and small intestine. Here we show that both lithium and valproate disrupt FXR signalling at the intestinal and hepatic levels, induce overproduction of BA in the liver and enhance both the circulating and the excreted BA pools. Given the important role of BAs and FXR signalling in regulating gastrointestinal physiology, as well as whole‐body lipid and glucose metabolism, these findings provide a foundation for further research to better understand the effects of lithium and valproate in human subjects.

## 2 METHODS

### 2.1 Ethics approval

Animal experiments were conducted under the European Directive 2010/63/EU and approved by the Animal Experimentation Ethics Committee of University College Cork (ethics no. 2016‐016, project authorisation AE19130/P049).

### 2.2 Animals and experimental groups

For the analysis of bile, protein and gene expression levels, we used plasma and tissue samples from rats that were chronically treated with either lithium or valproate (Cussotto et al., 2019). Briefly, adult male Sprague–Dawley rats (*n* = 8/group) were obtained from Envigo, Blackthorn, UK. They were housed two per cage, maintained under a 12‐h light–dark cycle and provided with chow and water ad libitum. Following 1 week of acclimatisation, animals received either (1) a standard rodent diet and drinking water (Vehicle group), (2) 0.2% lithium‐supplemented diet corresponding to approx. 150 mg/kg/day of lithium and hypertonic saline water (1.5% NaCl) to prevent lithium‐induced ionic imbalance (Lithium group), or (3) 2% valproate‐supplemented diet corresponding to approx. 1.5 g/kg/day of valproate (Valproate group). Rats in the same cage underwent the same treatment. Following 28 days of treatment, animals were euthanized by decapitation and tissue samples were collected and stored at −80°C for further analysis (Supporting information, Figure S1A). For the collection of plasma samples, trunk blood was centrifuged in the presence of K_2_EDTA at 3500 *g* for 10 min at +4°C, aliquoted and stored at −80°C for further analysis. For the follow‐up ex vivo studies on the acute effects of lithium and valproate on intestinal permeability and bile absorption, we used 11 adult male naïve Sprague–Dawley rats (approx. 300 g body weight) obtained from the same supplier and maintained in the same housing conditions as described above (Supporting information, Figure S1B,C).

### 2.3 Quantification of BA moieties in the in vivo and ex vivo studies

BAs and salts were measured as previously described (Joyce et al., 2014). Briefly, BAs were extracted following the addition of deuterated internal standards. Extracted BAs were resuspended in 50% methanol, injected in triplicate and assessed in negative electrospray mode through a C18 Acquity column using an LCT Premier quadrupole time of flight mass spectrometer (Waters, Wilmslow, UK). Assessment of extraction efficiency was performed using internal standards, and samples were quantified by standard curve construction for individual bile moieties using targetlynx software (Waters, Wilmslow, UK) (refer to Supporting information, Supplementary Methods for more details).

### 2.4 Gene expression analysis in the liver and distal ileum tissues

Total RNA was extracted from the distal ileum and the frontal lobe of the liver with the mirVana miRNA isolation kit (Thermo Fisher Scientific/Ambion, Waltham, MA, USA) following the manufacturer's protocol. A Nanodrop 1000 (Thermo Fisher Scientific, Waltham, MA, USA) was used to determine RNA concentration. RNA was reverse transcribed to cDNA using a high‐capacity cDNA reverse transcription kit (Thermo Fisher Scientific, Waltham, MA, USA) in a G‐storm thermocycler (G‐Storm, Somerton, UK). Genes of interest (Supporting information, Table S5) were amplified using SYBR Green primers and master mix (Thermo Fisher Scientific). Real‐time PCRs were run in triplicate on the LightCycler 480 instrument (Roche Diagnostics, Burgess Hill, UK). *C*_t_ values in triplicate were averaged and normalized to the *Actb* housekeeping gene. Data were analysed with the comparative cycle threshold method ($2^{-\Delta \Delta C_{t}}$) (Livak & Schmittgen, 2001) and presented as a fold change versus Vehicle group.

### 2.5 Functional analysis of caecal microbiota

The impact of lithium and valproate on the predicted function of the gastrointestinal microbial community was analysed using previously generated 16S rRNA sequencing data (Cussotto et al., 2019). Briefly, bacterial DNA was extracted from the caecal content using the Qiagen QIAmp Fast DNA Stool Mini Kit (Qiagen, Hilden, Germany) coupled with an initial bead‐beating step. The V3–V4 hypervariable region of the 16S rRNA gene was amplified and prepared for sequencing as outlined in the Illumina 16S Metagenomic Sequencing Library protocol. Samples were sequenced at Teagasc Sequencing Facility (Moorepark, Fermoy, Ireland) on the Illumina MiSeq platform using a 2 × 250 bp kit (Illumina, Inc., San Diego, CA, USA). Sequences were filtered based on a quality score threshold >30, trimmed, and filtered for quality and chimaeras using DADA2 (Callahan et al., 2016) in R (version 4.1.2). We used the amplicon sequence variants (ASVs) from DADA2 to infer function in terms of KEGG Orthologues (KOs) with PICRUSt2 (Douglas et al., 2020) using default settings. We then consulted the KEGG database (Kanehisa et al., 2016) and compiled a list of bile acid metabolism‐related functions by taking all KOs associated with primary and secondary bile acid biosynthesis and secretion (ko00120, ko00121 and ko04976), as well as any other KOs that mention bile acid. We cross‐referenced this list with the inferred KOs in our microbiome samples to assess alterations in the bile‐related metabolic potential of the microbiome. Functions with less than 10% prevalence were excluded from the analysis. Before all statistical testing, microbiome data were centred log‐ratio (clr) transformed. Custom R scripts are available at https://github.com/thomazbastiaanssen/Tjazi. A non‐parametric Kruskal–Wallis test followed by a pairwise Mann‐Whitney *U*‐test was used to assess differentially abundant functions. The Benjamini–Hochberg procedure was used to control the false discovery rate (FDR), with a *q*‐value threshold of 0.1.

### 2.6 Proteomic analysis in the liver and distal ileum tissues

Total protein was extracted, purified and prepared for mass spectrometry (MS) using iST Mammalian Tissue Sample Preparation Kit (PreOmics, Planegg, Germany) according to the manufacturer's protocol with minor modifications. For liquid chromatography–tandem mass spectrometry (LC‐MS/MS) analysis, samples were loaded onto individual EvoTips and run on a timsTOF Pro mass spectrometer (Bruker Daltonics, Germany) coupled to the EvoSep One system (Evosep BioSystems, Odense, Denmark) and operated in either Data Dependent Acquisition (DDA) or Data Independent Acquisition (DIA) mode. FragPipe (v19.1), with the DIA_SpecLib_Quant workflow selected, was used for database searching, protein identification and quantification by performing label‐free quantification of the identified peptides. For details, refer to Supporting information, Supplementary Methods.

The diann‐output.pg_matrix file containing normalized intensities for protein groups was used for subsequent statistical analysis in Rstudio GUI (version 2022.2.2.485). First, identified proteins were filtered to include proteins with >70% of valid values in at least one experimental group. Next, the intensity values were clr transformed using the Tjazi library (Bastiaanssen et al., 2023a; Bastiaanssen et al., 2023b). Zero count values were imputed using the ‘const’ method, taking two‐thirds of the lowest non‐zero sample value, as described by Lubbe et al. (2021). β‐Diversity was computed in terms of Aitchison distance or Euclidean distance between clr‐transformed data; principal component analysis (PCA) was used for visualisation. The differences in protein expression levels between groups were analysed using protein‐wise generalized linear models (GLMs). Dunnett's test with the Vehicle group as a control category was used *post hoc* to account for multiple comparisons. To correct for multiple testing, the Benjamini–Hochberg *post hoc* procedure was performed with a FDR *q*‐value of 0.05 as a cut‐off (Benjamini & Hochberg, 1995). Differentially abundant proteins with estimated effect sizes >0.5 and <−0.5 on the log2‐fold scale were selected for STRING pathway analysis https://string‐db.org/ (Szklarczyk et al., 2019). Plotting was handled using ggplot2. All R scripts are available online at https://github.com/thomazbastiaanssen/Tjazi (Bastiaanssen et al., 2023a; Bastiaanssen et al., 2023b).

### 2.7 Ex vivo assays of intestinal permeability and bile absorption

#### 2.7.1 Intestinal tissue preparation and mounting in Ussing chambers

Naïve animals were euthanized by decapitation, and freshly isolated distal ileum (8–10 cm segment adjacent to the caecum) was gently flushed and promptly stripped from the serosal and muscular layers on wet ice. The remaining tissue was mounted in Ussing chambers with an exposed tissue area of 0.126 cm^2^. Tissue was bathed in Krebs solution (in mM: 1.2 NaH_2_PO_4_, 117 NaCl, 4.8 KCl, 1.2 MgCl_2_, 25 NaHCO_3_, 11 CaCl_2_ and 10 glucose) at 37°C with continuous carbogen (95% O_2_, 5% CO_2_) supply. To exclude the impact of ileum Na^+^–glucose co‐transporter on the outcomes, glucose was replaced with 10 mM mannitol in the luminal chamber buffer.

#### 2.7.2 Macromolecular permeability and transepithelial electrical resistance (TEER)

To assess permeability to macromolecules, 4‐kDa fluorescein isothiocyanate (FITC)–dextran (Sigma, Merck, Arklow, Ireland) was added to the luminal chamber at a concentration of 2.5 mg/mL. Samples (200 µL) were collected from the serosal chamber at T0 (baseline), 30, 60, 90, 120, 150, and 180 min after the addition of FITC. FITC was quantified on a VICTOR‐1 plate reader (PerkinElmer, Waltham, MA, USA) at 485 nm excitation/535 nm emission wavelengths and the lumen‐to‐serosa total flux was presented in µg/h/cm^2^. To assess ionic flux, short‐circuit current (*I*_sc_) was continuously recorded in a zero‐voltage clamp mode and TEER was measured at each time point by discharging a 2 mV pulse. Tissues were treated with vehicle, lithium 5 mM, lithium 50 mM, valproate 10 mM or valproate 100 mM. Drugs were added to the luminal chamber at T0, one chamber per treatment in each animal (Supporting information, Figure S1B).

#### 2.7.3 Transepithelial absorption of BA

A bile acid mixture of known composition (pig bile spiked with rodent taurocholic acid (TCA) 600 ng/mL, taurodeoxycholic acid (TDCA) 120 ng/mL and tauromuricholic acid (TMCA) 330 ng/mL) was added to the luminal chamber at a dilution of 1:400. Following 180 min of incubation, samples (200 µL) were collected from the serosal chamber to estimate the transepithelial absorption of bile moieties. BAs were quantified as described above. Tissues were treated with vehicle, lithium 5 mM, lithium 50 mM, valproate 10 mM, valproate 100 mM or the ASBT inhibitor linerixibat 1 µM (GSK2330672, Cayman Chemical, Ann Arbor, MI, USA, CAS n.1345982‐69‐5). Drugs were added to the luminal chamber at T0, one chamber per treatment in each animal (Supporting information, Figure S1C).

### 2.8 Dose relevance for the ex vivo assays

To estimate the acute effects of lithium and valproate on intestinal permeability and bile absorption ex vivo, we deduced the concentrations of drugs in the distal ileum (site of reabsorption of BA) based on daily dose consumption and specific assumptions described in detail in Supporting information, Figure S2. Based on these approximations, 5 mM lithium and 10 mM valproate mimicked the concentrations in the rat distal ileum. To examine the dose–response relationship between drug concentration and physiological readouts, we assessed the same drugs in concentrations 10 times higher, that is, 50 mM lithium and 100 mM valproate.

### 2.9 Plasma levels of alanine aminotransferase, aspartate transaminase, fibroblast growth factor 19, cholesterol and triglycerides

Plasma concentrations of aspartate transaminase (AST) and alanine aminotransferase (ALT) were determined by enzyme‐linked immunosorbent assay (ELISA) according to the manufacturer's specifications (AST: MBS3809275, MyBioSource, San Diego, CA, USA; ALT: CSB‐E13024r, Cusabio, Houston, TX, USA). Assays sensitivity and intra‐/inter‐assay variability were, respectively, 2 U/L, coefficient of variation (CV) < 15% for AST and 0.78 U/L, CV < 10% for ALT. Plasma concentration of fibroblast growth factor 19 (Fgf19) was measured with Rat FGF19 ELISA Kit (LSBio, F23263, Newark, CA, USA). Plasma triglyceride levels were determined by using the EnzyChromTM Triglyceride Assay kit (ETGA‐200; BioAssay Systems, Hayward, CA, USA). Cholesterol was quantified with the Mouse Total cholesterol ELISA Kit (ab285242, Abcam, Cambridge, UK).

### 2.10 Statistical analysis

Data were assessed for normality and equality of variances using the Shapiro–Wilk test and Levene's test, respectively. For datasets with normal distribution and homogeneous variances, one‐way ANOVA followed by Dunnett's *post hoc* test was used. Non‐normally distributed datasets were analysed using the Kruskal–Wallis non‐parametric test followed by the Mann–Whitney *U*‐test with correction for multiple comparisons using the Benjamin–Hochberg FDR method. Grubbs's method was employed to test for outliers (Grubbs, 1950). The threshold for statistical significance was set at *P *< 0.05. Parametric data were presented as mean and SD, non‐parametric data as median with IQR and min‐to‐max values.

## 3 RESULTS

### 3.1 Lithium and valproate increase the bile acid pool in plasma

Primary BAs – cholic acid (CA) and chenodeoxycholic acid (CDCA) in humans; CA, CDCA and α‐ and β‐muricholic acids (α/β‐MCA) in rodents – are synthesized in the liver. The liver does not store bile; instead, BAs are conjugated with taurine or glycine and promptly released as salts from the liver into the intestine. In the intestine, primary bile salts are unconjugated from taurine and glycine and further metabolized by intestinal bacteria, yielding secondary and tertiary BAs (deoxycholic acid (DCA), lithocholic acid (LCA), ursodeoxycholic acid (UDCA), hyodeoxycholic acid (HDCA) and others). Both conjugated and unconjugated bile acid moieties are actively and passively reabsorbed from the intestine back into the circulation and in the liver, while the remaining bile is excreted with faeces. This so‐called enterohepatic circulation allows for the effective recycling of approximately 95% of bile moieties (reviewed in Perino et al., 2021). Unlike humans, rats do not have a gall bladder to store bile; hence, BAs in plasma represent the largest pool of bile moieties in rats, with a net balance between continuous secretion and reabsorption of bile.

**FIGURE 1:**
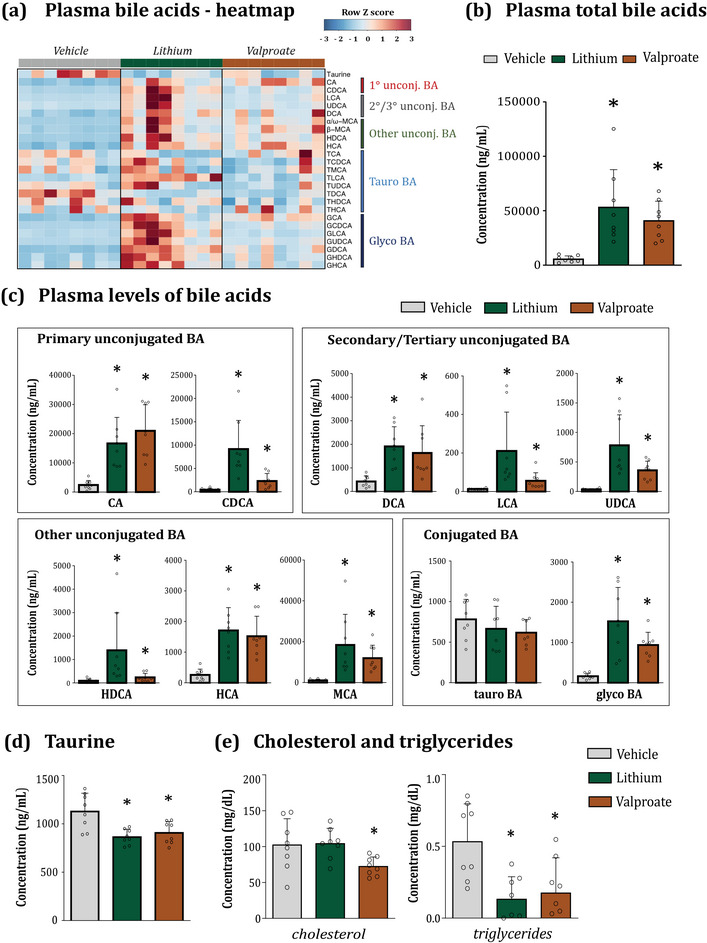
Lithium and valproate increase plasma bile acid pool. (a) Heatplot of plasma BA concentrations in vehicle‐, lithium‐ and valproate‐treated rats. BAs are shown along the *y*‐axis; individual animals are shown along the *x*‐axis. Spearman correlation of absolute quantified bile acid profile was performed. (b) Lithium and valproate increased total bile acid pool in plasma. (c) Both drugs increased concentrations of major bile moieties across all bile families. (d) Both drugs decreased taurine pool in plasma. (e) Effect of lithium and valproate on cholesterol and triglyceride levels in plasma. Statistical details: Data are presented as mean ± SD. ^*^ *P *< 0.05 (*n* = 7–8/group). (b) Total BA, KW test followed by MW test. KW χ^2^_(2) _= 15.48, *P *= 0.000, MW vs. lithium (Lit) *P *= 0.000, MW vs. valproate (Val) *P *= 0.000. (c) For CA, CDCA, DCA, LCA, UDCA, HDCA, MCA and glyco‐BA: KW test followed by MW. CA: KW χ^2^_(2) _= 15.86, *P *= 0.000, MW vs. Lit *P *= 0.000, MW vs. Val *P *= 0.000. CDCA: KW χ^2^_(2) _= 18.62, *P *= 0.000, MW vs. Lit *P *= 0.000, MW vs. Val *P *= 0.003. DCA: KW χ^2^_(2) _= 14.23, *P *= 0.000, MW vs. Lit *P *= 0.000, MW vs. Val *P *= 0.002. LCA: KW χ^2^_(2) _= 18.48, *P *= 0.000, MW vs. Lit *P *= 0.000, MW vs. Val *P *= 0.000. UDCA: KW χ^2^_(2) _= 16.64, *P *= 0.000, MW vs. Lit *P *= 0.000, MW vs. Val *P *= 0.000. HDCA: KW χ^2^_(2) _= 15.26, *P *= 0.000, MW vs. Lit *P *= 0.000, MW vs. Val *P *= 0.036. MCA: KW χ^2^_(2) _= 16.68, *P *= 0.000, MW vs. Lit *P *= 0.001, MW vs. Val *P *= 0.001. Glyco‐BA: KW χ^2^_(2) _= 16.08, *P *= 0.000, MW vs. Lit *P *= 0.000, MW vs. Val *P *= 0.000. For HCA, tauro‐BA: one‐way ANOVA followed by Dunnett's *post hoc* test. HCA: *F*_(2,23) _= 15.24, *P *= 0.000; vs. Lit *P *= 0.000, vs. Val *P *= 0.000. Tauro‐BA: *F*_(2,22) _= 0.99, *P *= 0.386. (d) Taurine: *F*_(2;23) _= 8.97, *P *= 0.002; vs. Lit *P *= 0.001, vs. Val *P *= 0.006. See also Supporting information, Table S1. (e) For cholesterol, triglycerides: one‐way ANOVA followed by Dunnett's *post hoc* test. Cholesterol: *F*_(2;23) _= 4.07, *P *= 0.032; vs. Lit *P *= 0.986, vs. Val *P *= 0.047. Triglycerides: *F*_(2;23) _= 7.54, *P *= 0.003; vs. Lit *P *= 0.004, vs. Val *P *= 0.009. BA, bile acids; CA, cholic acid; CDCA, chenodeoxycholic acid; DCA, deoxycholic acid; Glyco‐BA, glycine‐conjugated BA; HDCA, hyodeoxycholic acid; KW, Kruskal–Wallis; LCA, lithocholic acid; MCA, muricholic acid; MW, Mann–Whitney; Tauro‐BA, taurine‐conjugated BA; UDCA, ursodeoxycholic acid.

When we measured BA in plasma, we observed that both lithium and valproate induced a dramatic increase, exceeding 7‐fold in total bile acid levels (Figure 1b). This increase was primarily explained by the elevation of the most abundant primary BA: CA increased 8‐ to 10‐fold, CDCA 5‐ to 17‐fold, while MCA was elevated 10‐ to 12‐fold in the lithium and valproate groups, respectively (Figure 1a,c). This suggests a significant activation of bile acid synthesis in the liver. The levels of bacteria‐produced secondary and tertiary BAs (DCA, LCA, UDCA and HDCA) were also elevated in both groups (Figure 1a,c), indicating an activation of bacterial metabolism of bile due to the increased availability of the primary substrate.

Another interesting observation was noted regarding conjugated bile moieties. In rats, BAs are primarily conjugated with taurine rather than glycine (He et al., 2003; Subbiahet al., 1969), as seen in the Vehicle group, with a tauro‐BA:glyco‐BA ratio of approximately 5:1 (Figure 1c). However, this was not the case in the treatment groups. Valproate and, particularly, lithium substantially increased the concentration of glycine‐conjugated BAs, shifting the tauro‐BA:glyco‐BA ratio to 0.8:1 and 0.5:1, respectively. Interestingly, plasma levels of free taurine were significantly decreased in both treatment groups (Figure 1d). This suggests that the host production of taurine, in conjunction with its dietary intake, was likely insufficient to match the increased rates of bile synthesis in the liver. The glyco‐conjugation pathway must have been activated to compensate for this deficit, hence maintaining a high bile salts‐to‐BA ratio is critical for transporter‐mediated bile secretion.

Since primary BAs are produced from cholesterol, we also examined plasma levels of cholesterol and triglycerides (Figure 1e). Cholesterol was decreased by valproate treatment only, while triglyceride levels were reduced by both drugs. This points to alterations in lipid metabolism in the treatment groups, where a substantial portion of both exogenous and endogenous cholesterol would have been consumed for bile acid synthesis. Simultaneously, the concurrent enhanced energy demand would have increased β‐oxidation of free fatty acids and, thus, decreased systemic triglyceride levels.

### 3.2 Lithium and valproate increase BA in the liver

**FIGURE 2:**
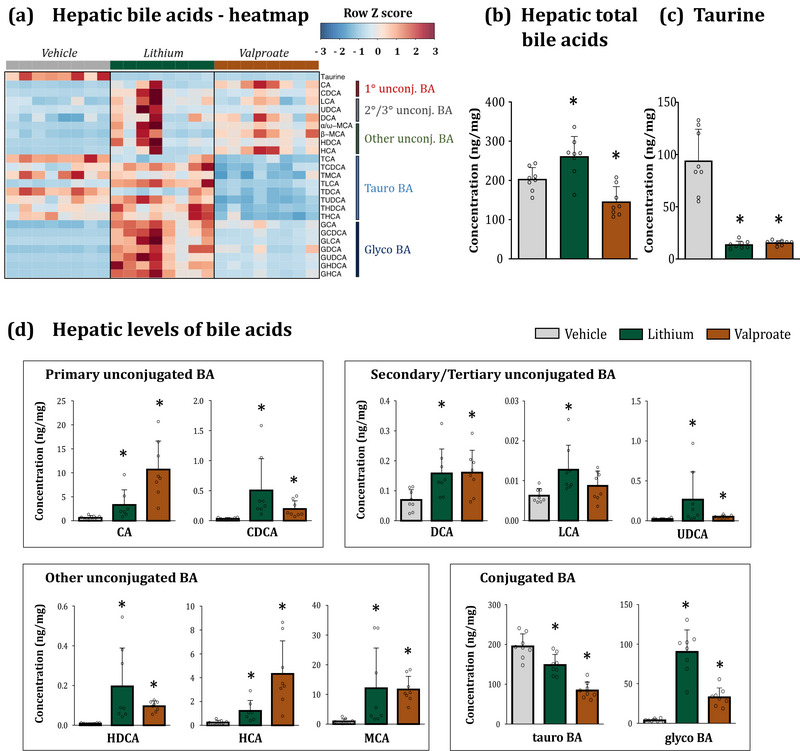
Lithium and valproate alter hepatic bile acid composition. (a) Heatmap of BA concentrations in the liver of vehicle‐, lithium‐ and valproate‐treated rats. BAs are shown along the *y*‐axis; individual animals are shown along the *x*‐axis. Spearman correlation of absolute quantified bile acid profile was performed. (b) Changes in total BA levels. (c) Lithium and valproate decreased taurine levels in the liver. (d) Both drugs increased concentrations of major bile moieties across all bile families. Statistical details: Data are presented as mean ± SD. ^*^ *P *< 0.05 (*n* = 7–8/group). (b) Total BAs, one‐way ANOVA followed by Dunnett's *post hoc* test. *F*_(2,23) _= 16.26, *P *= 0.000; vs. Lit *P *= 0.018, vs. Val *P *= 0.018. (c) Taurine, KW test followed by MW. KW χ^2^_(2) _= 16.34, *P *= 0.000, MW vs. Lit *P *= 0.001, MW vs. Val *P *= 0.001. (d) For CA, CDCA, UDCA, HDCA, HCA, MCA and glyco‐BA: KW test followed by MW. CA: KW χ^2^_(2) _= 16.46, *P *= 0.000, MW vs. Lit *P *= 0.005, MW vs. Val *P *= 0.001. CDCA: KW χ^2^_(2) _= 16.20, *P *= 0.000, MW vs. Lit *P *= 0.001, MW vs. Val *P *= 0.001. UDCA: KW χ^2^_(2) _= 12.29, *P *= 0.002, MW vs. Lit *P *= 0.003, MW vs. Val *P *= 0.005. HDCA: KW χ^2^_(2) _= 15.36, *P *= 0.000, MW vs. Lit *P *= 0.001, MW vs. Val *P *= 0.001. HCA: KW χ^2^_(2) _= 17.39, *P *= 0.000, MW vs. Lit *P *= 0.003, MW vs. Val *P *= 0.001. MCA: KW χ^2^_(2) _= 14.22, *P *= 0.002, MW vs. Lit *P *= 0.016, MW vs. Val *P *= 0.001. Glyco‐BA: KW χ^2^_(2) _= 18.89, *P *= 0.000, MW vs. Lit *P *= 0.001, MW vs. Val *P *= 0.001. For DCA, LCA and tauro‐BA: one‐way ANOVA followed by Dunnett's *post hoc* test. DCA: *F*_(2,23) _= 4.92, *P *= 0.018; vs. Lit *P *= 0.026, vs. Val *P *= 0.022. LCA: *F*_(2,23) _= 4.98, *P *= 0.017; vs. Lit *P *= 0.010, vs. Val *P *= 0.408. Tauro‐BA: *F*_(2,23) _= 35.8, *P *= 0.000; vs. Lit *P *= 0.004, vs. Val *P *= 0.000. See also Supporting information, Table S2. BA, bile acids; CA, cholic acid; CDCA, chenodeoxycholic acid; DCA, deoxycholic acid; Glyco‐BA, glycine‐conjugated BA; HDCA, hyodeoxycholic acid; KW, Kruskal–Wallis; LCA, lithocholic acid; MCA, muricholic acid; MW, Mann–Whitney; Tauro‐BA, taurine‐conjugated BA; UDCA, ursodeoxycholic acid.

Next, we analysed the effects of lithium and valproate on the bile acid pool in the liver, the site of bile synthesis. As previously mentioned, hepatocytes promptly excrete bile into the bile ducts following conjugation with amino acids, and thus the hepatic bile represents only a small fraction of the total bile acid pool. Consistent with the findings in plasma, we observed a substantial increase in unconjugated primary bile acid levels (CA, CDCA and MCA), as well as in secondary and tertiary bile aids (DCA, LCA, UDCA and HDCA) in both treatment groups (Figure 2a,d). Furthermore, aligning with the observations in plasma samples, we noted a significant suppression of tauro‐conjugation with a concurrent activation of glyco‐conjugation of BAs (Figure 2d). Hepatic taurine levels were also markedly decreased (Figure 2c). Given that conjugated BAs represent the largest fraction of bile moieties in the liver, changes in conjugation rates were likely to explain the observed alterations in total BA levels. In the lithium group, a modest decrease in tauro‐BA was compensated by a >20‐fold increase in glyco‐BA, resulting in the elevation of the total bile hepatic pool. Interestingly, in the valproate group, a 50% reduction in tauro‐BA was not fully compensated by a lesser increase in glyco‐BA, leading to a net reduction in the total BA pool (Figure 2b).

### 3.3 Lithium and valproate increase faecal excretion of bile moieties

**FIGURE 3:**
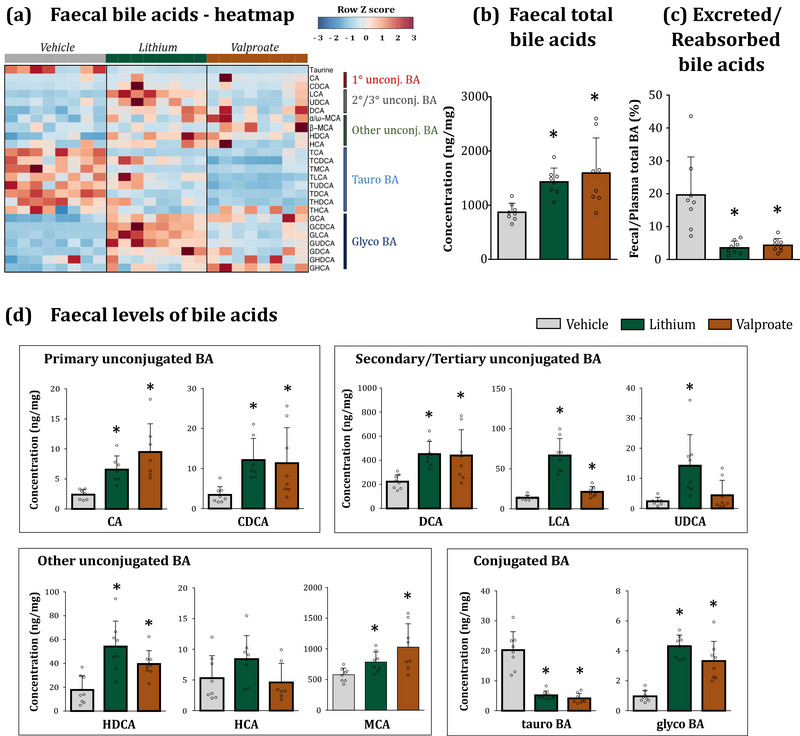
Lithium and valproate increase faecal bile acid pool. (a) Heatplot of faecal BA concentrations in vehicle‐, lithium‐ and valproate‐treated rats. BAs are shown along the *y*‐axis; individual animals are shown along the *x*‐axis. Spearman correlation of absolute quantified bile acid profile was performed. (b) Lithium and valproate increased total BA levels in the faeces. (c) Both drugs decreased the excreted‐to‐reabsorbed bile ratio. (d) Both drugs altered concentrations of major bile moieties across all bile families. Statistical details: data are presented as mean ± SD. ^*^ *P *< 0.05 (*n* = 7–8/group), KW test followed by MW test. (b) Total BA: KW χ^2^_(2) _= 12.18, *P *= 0.002, MW vs. Lit *P *= 0.001, MW vs. Val *P *= 0.006. (c) Excreted/reabsorbed ratio: KW χ^2^_(2) _= 15.17, *P *< 0.001, MW vs. Lit *P *= 0.001, MW vs. Val *P *= 0.006. (d) CA: KW χ^2^_(2) _= 14.52, *P *= 0.000, MW vs Lit *P *= 0.002, MW vs Val *P *= 0.002. CDCA: KW χ^2^_(2) _= 12.32, *P *= 0.002, MW vs Lit *P *= 0.001, MW vs. Val *P *= 0.009. DCA: KW χ^2^_(2) _= 11.8, *P *= 0.003, MW vs. Lit *P *= 0.000, MW vs. Val *P *= 0.016. LCA: KW χ^2^_(2) _= 17.01, *P *= 0.000, MW vs. Lit *P *= 0.000, MW vs. Val *P *= 0.028. UDCA: KW χ^2^_(2) _= 10.87, *P *= 0.004, MW vs. Lit *P *= 0.002, MW vs. Val *P *= 0.908. HDCA: KW χ^2^_(2) _= 12.7, *P *= 0.002, MW vs. Lit *P *= 0.003, MW vs. Val *P *= 0.005. HCA: KW χ^2^_(2) _= 4.87, *P *= 0.087. MCA: KW χ^2^_(2) _= 16.81, *P *= 0.000, MW vs. Lit *P *= 0.000, MW vs. Val *P *= 0.000. Tauro‐BA: KW χ^2^_(2) _= 16.2, *P *= 0.000, MW vs. Lit *P *= 0.000, MW vs. Val *P *= 0.000. Glyco‐BA: KW χ^2^_(2) _= 16.64, *P *= 0.000, MW vs. Lit *P *= 0.000, MW vs. Val *P *= 0.000. See also Supporting information, Table S3. BA, bile acids; KW, Kruskal–Wallis; MW, Mann–Whitney.

Next, we examined the gastrointestinal tract as a site for bile reabsorption, bacterial metabolism of bile, and bile excretion. Initially, we assessed the bile acid pool in faecal matter, which represents the excreted bile components. Both treatments resulted in increased total bile acid levels in the faeces: a 1.6‐fold increase in the lithium group and a 1.8‐fold increase in the valproate group (Figure 3b). This is a significant but not extensive loss of bile. For comparison, mice deficient in the intestinal ASBT transporter, which mediates active absorption of bile, display an over 10‐fold elevation of excreted bile in their faeces (Dawson et al., 2003).

The observed increase primarily stemmed from elevated excretion of the most abundant unconjugated secondary BAs – DCA, LCA and HDCA – which saw a significant increase of 2‐ to 5‐fold, particularly evident in the lithium group (Figure 3a,d). The levels of primary BAs (CA, CDCA and MCA) were also elevated in both treatment groups. The elevation in the secondary bile acid pool likely occurred due to the activation of bacterial metabolism. As mentioned earlier, in the lower intestine, BAs undergo transformation by the resident microbiota (reviewed in Long et al., 2017; Wahlstrom et al., 2016). Initially, bacterial bile salt hydrolase activity deconjugates bile moieties from taurine and glycine, and these can be further transformed into secondary and tertiary BAs via several pathways including 7α‐hydroxysteroid dehydrogenase activity (Tian et al., 2020) (Supporting information, Figure S3A). This bacteria‐mediated transformation of bile is crucial as secondary BAs possess a distinct signalling profile in the gastrointestinal tract, influencing gut motility, secretion and epithelial barrier function (Hegyi et al., 2018).

The percentage of conjugated bile excreted in faeces in the treatment groups was comparable to the vehicle control: 0.5–0.6% in the lithium and valproate groups versus 2.4% in the vehicle group, indicating an adequate bacterial deconjugation rate (see Supporting information, Table S3). Furthermore, upon analysing the impact of lithium and valproate on the predicted function of gut bacteria using previously generated 16S rRNA sequencing data (Cussotto et al., 2019), we noted a significant increase in 7α‐hydroxysteroid dehydrogenase function (EC: 1.1.1.159) in both treatment groups. Additionally, the valproate group displayed an increase in 7β‐hydroxy‐3‐oxochol‐24‐oyl‐CoA 4‐desaturase function (EC: 1.3.1.116) (Supporting information, Figure S3B). These enzymes are part of the bacterial secondary bile acid biosynthesis pathway. These findings indicate that the gut microbiome in lithium‐ and valproate‐treated animals adapted to the increased bile flux in the intestine by activating bile‐metabolizing capacities as a compensatory response.

In summary, the analysis of bile acid composition in plasma, liver, and faeces revealed that chronic administration of lithium and valproate led to sustained activation of bile acid synthesis in the liver. This activation was accompanied by an elevation of the bile acid pool in the systemic circulation, activation of bacteria‐mediated bile metabolism and increased faecal excretion of bile components. To comprehend the underlying molecular changes, we conducted hepatic proteome profiling to investigate the effects of lithium and valproate on liver metabolism in general and specifically on bile metabolic pathways. Changes in bile‐associated proteins were also confirmed at the gene expression level.

### 3.4 Lithium and valproate induce substantial up‐regulation of bile acid synthesis in the liver

**FIGURE 4:**
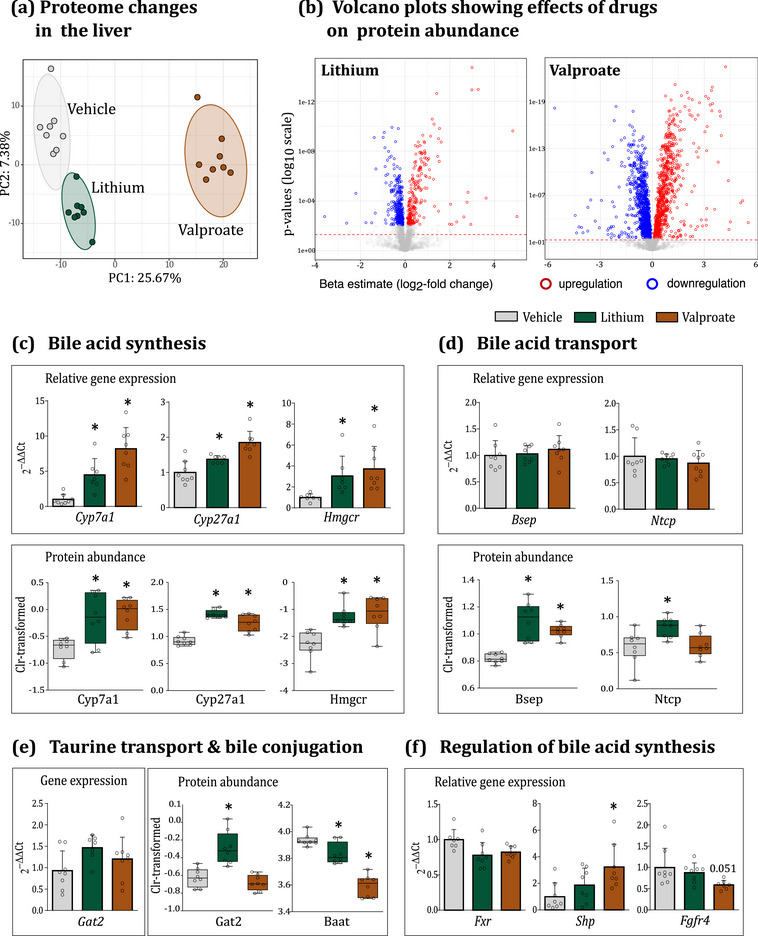
Lithium and valproate disrupt FXR/SHP signalling and induce sustained up‐regulation of Cyp7a1 and Cyp27a in the liver. (a) PCA of proteomics data revealed a significant impact of both drugs on the hepatic proteome. (b) Volcano plots showing hepatic proteins differentially expressed following treatment with lithium (left) and valproate (right panel). The *x*‐axis represents the fold change in protein expression between the vehicle and the respective treatment groups (β), and the *y*‐axis depicts *P*‐values. Red and blue points represent proteins that were significantly up‐ and down‐regulated, respectively, following FDR filtering at a 0.05 cut‐off. Dashed horizontal lines represent the pre‐adjusted *P* = 0.05 threshold. (c–f) Protein and gene expression data of key enzymes, transporters and transcription factors implicated in the hepatic bile acid metabolism. For the transcription factors, only gene expression data were available. Due to the low abundance of these proteins, we could not detect them with the LC/MS method. Statistical details for (c–f): Data are presented as mean ± SD and as median and IQR and min‐to‐max values. ^*^ *P *< 0.05 (*n* = 7–8/group). For protein data, GLM followed by Dunnett's *post hoc* test. Cyp7a1: *F*_(2,24) _= 9.09, *P *= 0.001; vs. Lit *P *= 0.004, vs. Val *P *= 0.002. Cyp27a1: *F*_(2,24) _= 41.44, *P *= 0.000; vs. Lit *P *= 0.000, vs. Val *P *= 0.000. Hmgcr: *F*_(2,24) _= 11.94, *P *= 0.000; vs. Lit *P *= 0.001, vs. Val *P *= 0.001. Bsep: *F*_(2,24) _= 21.99, *P *= 0.000; vs. Lit *P *= 0.000, vs. Val *P *= 0.000. Ntcp: *F*_(2,24) _= 5.78, *P *= 0.01; vs. Lit *P *= 0.011, vs. Val *P *= 0.959. Gat2: *F*_(2,24) _= 19.96, *P *= 0.000; vs. Lit *P *= 0.000, vs. Val *P *= 0.589. Baat: *F*_(2,24) _= 49.86, *P *= 0.000; vs. Lit *P *= 0.015, vs. Val *P *= 0.000. For gene expression data, *Cyp7a1* and *Hmgcr*: KW test followed by MW. *Cyp7a1*: KW χ^2^_(2) _= 31.74, *P *= 0.000, MW vs. Lit *P *= 0.002, MW vs. Val *P *= 0.001. *Hmgcr*: KW χ^2^_(2) _= 13.45, *P *= 0.001, MW vs. Lit *P *= 0.014, MW vs. Val *P *= 0.002. *Cyp27a1*, *Bsep*, *Ntcp*, *Gat2*, *Fxr*, *Shp*, *Fgfr4*: one‐way ANOVA followed by Dunnett's *post hoc* test. *Cyp27a1*: *F*_(2,22) _= 19.62, *P *= 0.000; vs. Lit *P *= 0.028, vs. Val *P *= 0.000. *Bsep*: *F*_(2,23) _= 0.49, *P *= 0.617. *Ntcp*: *F*_(2,23) _= 0.54, *P *= 0.59. *Gat2*: *F*_(2,22) _= 2.76, *P *= 0.087. *Fxr*: *F*_(2,23) _= 12.07, *P *= 0.000; vs Lit *P *= 0.161, vs. Val *P *= 0.344. *Shp*: *F*_(2,23) _= 2.35, *P *= 0.045; vs. Lit *P *= 0.655, vs. Val *P *= 0.013. *Fgfr4*: *F*_(2,23) _= 3.48, *P *= 0.051; vs. Lit *P *= 0.647, vs. Val *P *= 0.033. BA, bile acids; FXR, farnesoid X receptor; KW, Kruskal–Wallis; MW, Mann–Whitney; PCA, principal component analysis; SHP, small heterodimer partner.

Approximately 3300 proteins were identified in liver tissues from the vehicle‐, lithium‐ and valproate‐treated animals for differential expression profiling (Supporting information, Table S6 and Figures S4, S5). PCA showed significant changes in the hepatic proteome caused by both drugs, particularly pronounced with valproate (Figure 4a). Comparing individual protein expression between vehicle and drug‐treated animals revealed 547 and 1839 significantly changed proteins in the lithium and valproate groups, respectively, with a comparable proportion of up‐regulated and down‐regulated proteins (as depicted in volcano plots in Figure 4b). To gain an overview of metabolic pathways and protein classes affected by drugs, we used differentially abundant proteins with log2 effect size >0.5 and <−0.5 as an input for protein network and functional enrichment analysis using the STRING database (Szklarczyk et al., 2019). Valproate prominently affected proteins associated with mitochondria, that is, mitochondrial metabolism, structure, transport, oxidative phosphorylation, and fatty acid β‐oxidation (Supporting information, Figure S5). Conversely, chronic lithium administration up‐regulated cholesterol and bile acid synthetic pathways, alongside activation of peroxisomal fatty acid oxidation (Supporting information, Figure S4). Further details on the affected metabolic pathways and potential underlying mechanisms are elaborated in the supporting information (Supporting information, Figures S4, S5). It is worth noting that peroxisomes play a crucial role in both bile acid synthesis and conjugation (Ferdinandusse et al., 2009).

**FIGURE 5:**
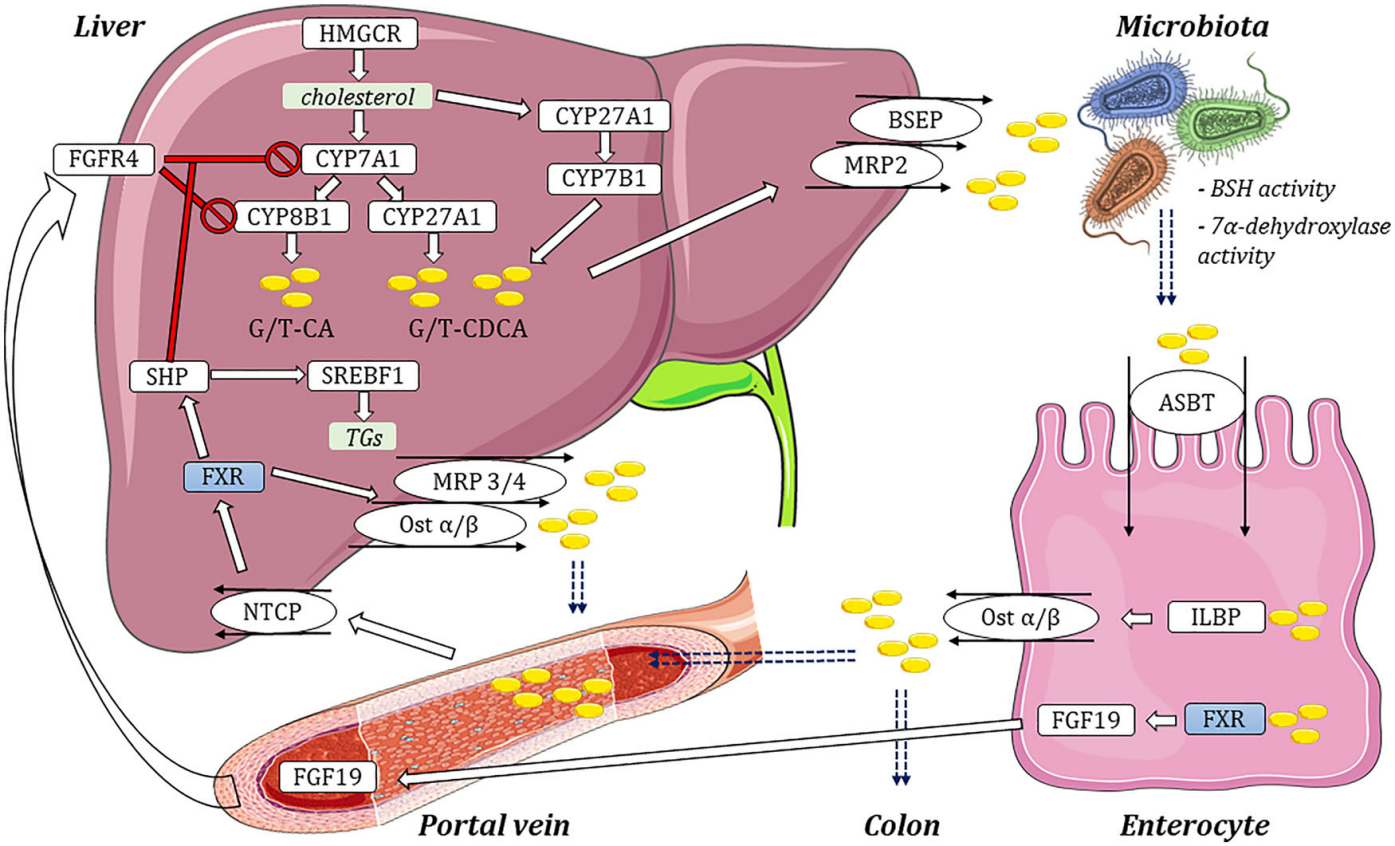
Schematic of hepatic synthesis and enterohepatic circulation of BAs. Yellow circles indicate conjugated bile salts, blue dashed arrows indicate the movement of bile moieties to different body compartments, white arrows indicate the direction of signalling and red arrows indicate negative feedback signalling. ASBT, apical sodium–bile acid transporter; BSEP, bile salt export pump; BSH, bile salt hydrolase; CYP, cytochrome P450; FGF19, fibroblast growth factor 19; FGFR4, fibroblast growth factor receptor 4; FXR, farnesoid X receptor; G/T‐CA, glyco/tauro‐cholic acid; G/T‐CDCA, glyco/tauro‐chenodeoxycholic acid; HMGCR‐3, hydroxy‐3‐methylglutaryl‐CoA reductase; ILBP, ileal lipid‐binding protein; MRP, multidrug resistance protein; NTCP, Na^+^–taurocholate cotransporting polypeptide; Ost α/β, organic solute transporter α/β; SHP, small heterodimer partner; SREBF1, sterol regulatory element‐binding transcription factor 1; TGs, triglycerides.

We further looked into the individual proteins involved in bile metabolic pathways. Primary BAs are synthesized in the liver from cholesterol via classical and alternative pathways initiated by cytochrome P450 (CYP) 7A1 and CYP27A1 enzymes, respectively (Figure 5) (Russell, 2003). Bile acid synthesis is finely regulated by a negative feedback mechanism wherein bile moieties can suppress their synthesis through hepatic and intestinal FXR signalling (Rizzo et al., 2005; Somm & Jornayvaz, 2018). FXR is a highly specific bile acid receptor that is primarily activated by CDCA, DCA and LCA (Parks et al., 1999). Elevated hepatic bile acid concentrations activate FXR, leading to the transcription of its downstream small heterodimer partner (SHP). SHP, in turn, down‐regulates CYP7A1 and CYP8B1 expression, suppressing bile acid synthesis. Simultaneously, an increase in cholesterol levels, a bile acid precursor, down‐regulates 3‐hydroxy‐3‐methylglutaryl‐CoA reductase (HMGCR) expression – the rate‐limiting enzyme in cholesterol synthesis – via SREBP2 signalling (Luo et al., 2020).

However, in lithium‐ and valproate‐treated animals, Cyp7a1, Cyp27a1 and Hmgcr were markedly up‐regulated at both protein and gene levels indicating activation of bile and cholesterol biosynthesis (Figure 4c). The *Fxr* gene remained unaffected by either drug, while *Shp* showed significant up‐regulation in the valproate group only (Figure 4f). We also looked at the expression of the hepatic bile transporters regulated by FXR (reviewed in Wagner et al., 2010). The ‘influx’ NTCP (SLC10A1) transporter, which is responsible for bile uptake from the portal blood into hepatocytes, is down‐regulated by the FXR/SHP pathway (Denson et al., 2001). The major ‘efflux’ transporters – BSEP (ABCB11, excretes bile salts into the bile ducts) and MRP3/4 (ABCC3/4, transport bile to the sinusoidal blood) – are up‐regulated by FXR either directly or indirectly (Halilbasic et al., 2013; Plass et al., 2002). All these measures aim to protect the liver from bile overload. Bsep and Mrp3/4 transporters were up‐regulated in both treatment groups (Figure 4d and Supporting information, Figure S6C). However, Ntcp transporter remained unchanged in the valproate group but, surprisingly, was up‐regulated in the lithium group (Figure 4d).

Considering the depletion of plasma and hepatic taurine levels in drug‐treated animals (Figures 1d and 2c), we examined hepatic taurine transporters. Gat2 (Slc6a13), the primary transporter of taurine into the liver in rodents (Kubo et al., 2016), displayed up‐regulation in the lithium group, likely due to the loss of inhibitory control from circulating taurine (Zhou et al., 2012). However, Gat2 levels remained unchanged in the valproate group (Figure 4e). The gene expression of the minor hepatic taurine transporter TauT (Slc6a6) was down‐regulated by both drugs (Supporting information, Figure S6C). The expression of bile acid‐CoA:amino acid *N*‐acyltransferase (Baat), a peroxisomal enzyme catalysing the transfer of bile acyl‐CoA thioester to either glycine or taurine, was down‐regulated by both drugs, particularly impacted by valproate (Figure 4e). These findings align with alterations in hepatic tauro‐bile levels, where valproate showed marked suppression of tauro‐conjugation and lesser activation of the glyco‐conjugation pathway (Figure 2d).

In summary, these findings indicate a malfunction in FXR‐mediated regulation of bile acid synthesis and transport in treated animals. Despite significantly increased circulating levels of BAs, the FXR pathway failed to shut down bile acid synthesis, resulting in substantial bile overproduction. Each drug had a unique impact on FXR signalling. Lithium seemed to have targeted FXR's ability to induce Shp expression. While *Shp* gene expression remained unchanged in the lithium group, the inhibitory effects of Shp on Cyp7a1 and Ntcp expression were reversed by lithium, resulting in a substantial up‐regulation of both proteins (Figure 4c,d). In contrast, in the valproate group, the up‐regulation of *Shp* expression suggested that the drug interfered downstream, blocking the Shp‐mediated suppression of Cyp7a1 (which was up‐regulated) and Ntcp (with no change, Figure 4c,d).

We questioned whether hepatic inflammation or cellular toxicity might be involved. Elevated serum bile acid levels have been linked to liver injury (Luo et al., 2013; Luo et al., 2018). Exposure of hepatocytes to increased concentrations of bile compounds can provoke liver inflammation (Allen et al., 2011; Li et al., 2017). These pathophysiological changes could have potentially desensitised the FXR regulatory pathway. Thus, we assessed the gene expression of pro‐inflammatory and pro‐fibrosis mediators in the liver (Supporting information, Figure S6A). However, the expression levels of all target genes were consistent across the groups. Additionally, the plasma activities of ALT and AST, systemic markers of liver damage, showed no significant alterations in the drug‐treated groups (Supporting information, Figure S6B). Hence, we found no evidence of inflammatory response or tissue damage in the livers of lithium‐ or valproate‐treated animals.

We then hypothesised that changes in hepatic bile acid transport might have contributed to FXR signalling malfunction. Indeed, the plasma‐to‐liver ratio for total BA rose from 28:1 in the vehicle group to 165:1 and 280:1 in the lithium and valproate groups, respectively, indicating reduced hepatic clearance of bile moieties (Figures 1, 2). Although the hepatic levels of CDCA, LCA and DCA, the most potent FXR activators (Parks et al., 1999), were elevated in the lithium group, they were decreased in the valproate group (22.2, 47.5 and 13.6 ng/mg in the vehicle, lithium and valproate groups, respectively, both free and conjugated forms, Supporting information, Table S2). This suggests that a deficit in bile acid uptake might have impacted the efficacy of FXR activation, at least in valproate‐treated animals. On the other hand, increased rates of bile acid ‘efflux’ (due to the up‐regulation of Bsep and Mrp3/4 transporters, Figure 4d and Supporting information, Figure S6C) could have masked the true rates of hepatic bile uptake.

It is important to note that hepatic regulation of bile metabolism, especially in conjunction with lipid and glucose metabolism, involves numerous regulatory elements, including RXR, PXR, CAR, HNF4, MAFG and others (Chiang, 2015; Halilbasic et al., 2013; Perino et al., 2021). Furthermore, bile acid synthesis in the liver is regulated not only via the hepatic FXR/SHP‐mediated pathway but also through the intestinal FXR/FGF19 negative feedback mechanism (Figure 5, reviewed in Somm & Jornayvaz, 2018). In distal ileum, bile salts are actively absorbed from the gut lumen through the ASBT (SLC10A2) transporter (Dawson et al., 2003; Dawson & Karpen, 2015). In enterocytes, bile salts activate the FXR receptor and up‐regulate the expression of its downstream target, FGF15/19, a potent inhibitor of bile synthesis. FGF15/19 enters the circulation, travels to the liver, activates its receptor fibroblast growth factor receptor 4 (FGFR4) and down‐regulates the expression of CYP7A1 and CYP7B1 genes via βKlotho/c‐Jun N‐terminal kinase (JNK)/extracellular signal‐regulated kinase (ERK) pathways (Figure 5; Holt et al., 2003; Inagaki et al., 2005; Kong et al., 2012). In rodents, evidence suggests that the intestinal Fxr/Fgf19 pathway plays a critical role in suppressing *Cyp7a1* gene expression, while the hepatic Fxr/Shp pathway has a minor impact on the *Cyp7a1* gene (Kong et al., 2012). To better comprehend the mechanisms behind hepatic changes in treated animals, we analysed the effects of lithium and valproate on bile acid transport and FXR signalling in the small intestine.

### 3.5 Effects of lithium and valproate on the Fxr/Fgf19 pathway and bile absorption in the small intestine

**FIGURE 6:**
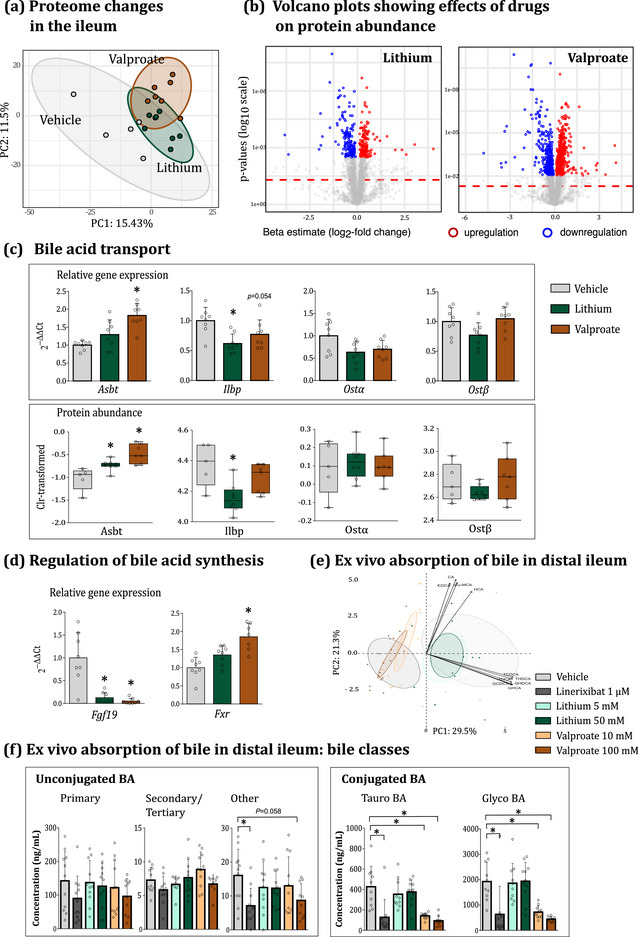
Effects of lithium and valproate on FXR signalling and active bile absorption in distal ileum. (a) PCA revealed a significant impact of both drugs on the proteome in ileum tissue. (b) Volcano plots showing individual proteins affected by lithium (left) and by valproate (right panel) in distal ileum. The *x*‐axis represents the estimated difference in means between the vehicle and the respective treatment groups (β), and the *y*‐axis depicts *P*‐values on a log10 scale. Red and blue points represent proteins that were significantly up‐ and down‐regulated, respectively, following FDR filtering at a 0.05 cut‐off. Dashed horizontal line represents the pre‐adjusted *P* = 0.05 threshold. (c) Protein and gene expression data of key bile transporters. (d) Gene expression of Fxr/Fgf19 factors. Proteins for these genes were not detected using LC/MS/MS proteomic analysis. (e–f) Acute effects of lithium, valproate and ASBT inhibitor linerixibat on bile absorption in distal ileum. Tissues were mounted into Ussing chambers and treated with drugs in the luminal (epithelial) compartment. Reabsorbed bile moieties were analysed in the serosal compartment at 180 min following drug administration. (e) PCA showed significant impact of linerixibat and valproate on the composition of the absorbed bile moieties. Lithium had no effect on bile absorption. Data were centred and scaled, then plotted with the function fviz_pca_biplot (factoextra package), choosing ellipse.level = 0.95 and ellipse.type = ‘confidence’. (f) Between‐group comparisons of individual bile moieties revealed that linerixibat and valproate significantly impaired the absorption of conjugated BA (tauro‐ and glyco‐bile salts). Statistical details for (c, d): data are presented as means ± SD and as median and IQR and min‐to‐max values. ^*^ *P *< 0.05 (*n* = 7–8/group). For protein data, GLM followed by Dunnett's *post hoc* test. Asbt: *F*_(2,20) _= 12.62, *P *= 0.000; vs. Lit *P *= 0.024, vs. Val *P *= 0.000. Ilbp: *F*_(2,20) _= 6.93, *P *= 0.006; vs. Lit *P *= 0.004, vs. Val *P *= 0.242. Ostα: *F*_(2,20) _= 0.14, *P *= 0.869. Ostβ: *F*_(2,20) _= 1.51, *P *= 0.249. For gene expression data, Fxr, Ilbp, Ostβ, Asbt: one‐way ANOVA followed by Dunnett's *post hoc* test. Fxr: *F*_(2,22) _= 7.51, *P *= 0.000; vs. Lit *P *= 0.154, vs. Val *P *= 0.000. Ilbp: *F*_(2,22) _= 6.62, *P *= 0.000; vs. Lit *P *= 0.000, vs. Val *P *= 0.054. Ostβ: *F*_(2,23) _= 2.07, *P *= 0.073. Asbt: *F*_(2,23) _= 14.34, *P *= 0.000; vs. Lit *P *= 0.128, vs. Val *P *= 0.000. Fgf19 and Ostα: KW test followed by MW. Fgf19: KW χ^2^_(2) _= 38.74, *P *= 0.000, MW vs. Lit *P *= 0.002, MW vs. Val *P *= 0.002. Ostα: KW χ^2^_(2) _= 14.52, *P *= 0.024, MW vs. Lit *P *= 0.074, MW vs. Val *P *= 0.093. Statistical details for (f): data are presented as means ± SEM. ^*^ *P *< 0.05 (*n* = 10–11/group). For tauro‐BA, glyco‐BA: KW test followed by MW. Tauro‐BA: KW χ^2^_(5) _= 37.41, *P *= 0.000, vs. Lin *P *= 0.003, vs. Lit5 *P *= 0.496, vs. Lit50 *P *= 0.673, vs. Val10 *P *= 0.001, vs. Val100 *P *= 0.000. Glyco‐BA: KW χ^2^_(5) _= 38.82, *P *= 0.000, vs. Lin *P *= 0.003, vs. Lit5 *P *= 0.821, vs. Lit50 *P *= 0.778, vs. Val10 *P *= 0.001, vs. Val100 *P *= 0.000. For unconjugated BA: one‐way ANOVA followed by Dunnett's *post hoc* test. Primary unconj. BA: *F*_(5,64) _= 0.986, *P *= 0.434. Secondary/tertiary unconj. BA: *F*_(5,62) _= 2.75, *P *= 0.027; vs. Lin *P *= 0.338, vs. Lit5 *P *= 0.932, vs. Lit50 *P *= 0.993, vs. Val10 *P *= 0.274, vs. Val100 *P *= 0.956. Other unconj. BA: *F*_(5,64) _= 2.51, *P *= 0.040; vs. Lin *P *= 0.012, vs. Lit5 *P *= 0.601, vs. Lit50 *P *= 0.554, vs. Val10 *P *= 0.729, vs. Val100 *P *= 0.058. FXR, farnesoid X receptor; PCA, principal component analysis.

We conducted proteome profiling in distal ileum tissues of chronically treated animals. Changes in proteins of interest were further validated at the gene expression level. Over 4600 proteins were identified in ileum tissues from the vehicle‐, lithium‐ and valproate‐treated animals (Supporting information, Table S7). PCA showed a significant impact of both drugs on protein abundances (Figure 6a): 301 and 844 differentially abundant proteins were found in the lithium and valproate groups, respectively, with a comparable proportion of up‐regulated and down‐regulated proteins (as illustrated in volcano plots in Figure 6b). Interestingly, the impact of both treatments on the intestinal proteome was relatively minor compared to the liver (Supporting information, Figures S4, S5 and Table S6). In line with this observation, the STRING pathway analysis did not reveal distinctive large clusters of affected protein pathways in the ileum of either lithium or valproate group (Supporting information, Figures S7, S8).

Subsequently, we focused specifically on proteins involved in bile transport and FXR signalling in these samples (Figure 6c,d). When BA levels in the intestinal lumen rise, they activate the FXR receptor in enterocytes and down‐regulate the expression of bile transporters – ASBT (import of bile salts from the lumen into the enterocytes), ileal lipid‐binding protein (ILBP; intracellular transport of bile moieties) and organic solute transporter α/β (OSTα/β; export of bile from the enterocytes into the circulation) – to restrict the absorption of excessive bile and normalise the BA plasma pool (Halilbasic et al., 2013; Ticho et al., 2019). Most importantly, activation of FXR receptor up‐regulates the expression of FGF19 to suppress hepatic bile synthesis (Kong et al., 2012).

However, this regulation was disrupted in lithium‐ and valproate‐treated animals. Despite a substantial increase in the BA pool, the expression of the *Fxr* gene was up‐regulated by valproate only (Figure 6d). The expression of the *Fgf19* gene was dramatically suppressed by both drugs. Circulating levels of Fgf19 were also reduced in both treatment groups (Supporting information, Figure S9A), although this difference was not significant due to the high variability of data in the vehicle group. Asbt levels were up‐regulated by both drugs, Ostα/β expression was not affected, while Ilbp levels were down‐regulated (Figure 6c).

In agreement with the liver data, these findings indicate a malfunction in FXR‐mediated signalling in treated animals, with the Fgf19 pathway being particularly affected. We also examined the expression of *Fgfr4* gene – the receptor of Fgf15/19 – in the liver and it was marginally affected by either drug (*P* = 0.051, one‐way ANOVA, Figure 4f). Hence, it must be the deficit in the activation of intestinal FXR/FGF19 negative feedback axis that could have contributed to the sustained activation of the hepatic Cyp7a1 expression and BA synthesis in the treated animal. We questioned whether the deficit in BA absorption or the direct interference of drugs with FXR signalling could be the cause of these changes.

While we did not directly measure the BA pool in the small intestine, the data collected in our study suggest adequate absorption of bile in drug‐treated animals. First, despite a >7‐fold elevation of plasma (reabsorbed) BA pool (Figure 1b), lithium‐ and valproate‐treated animals displayed only a 1.6‐ to 1.8‐fold elevation of faecal (excreted) BA pool (Figure 3b). Thus, the faeces‐to‐plasma bile ratio (i.e., percentage of lost bile) decreased from about 20% in the vehicle group to 3–4% in both treatment groups (Figure 3c). Second, acute ex vivo treatment of ileum tissues with 5 mM lithium or 10 mM valproate did not cause any significant damage to the intestinal epithelium, as shown by the absence of changes in epithelial tight junction integrity (TEER), secretory activity (*I*_sc_), as well as macromolecular permeability to 4 kDa FITC (Supporting information, Figure S9C, D). Chronic administration of lithium or valproate similarly did not affect FITC intestinal permeability (Cussotto et al., 2019), nor did it induce activation of pro‐inflammatory cytokines in the distal ileum (Supporting information, Figure S9B).

Lastly, it should be noted that bile in the intestine can be reabsorbed via both active and passive routes (Krag & Phillips, 1974; Schiff et al., 1972). The active transport of bile salts is mediated via the ASBT transporter. Unconjugated bile moieties, in their protonated form, are further absorbed via passive ionic diffusion down the chemical gradient (Dawson & Karpen, 2015). We observed that both drugs up‐regulated the expression of Asbt transporter (Figure 6c), indicating a potential activation of active bile absorption. Hence, we measured the BA uptake in the small intestine to investigate whether up‐regulation of active bile transport could have contributed to the observed reduction in faeces‐to‐plasma bile ratios in treatment groups (Figure 3c). Distal ileum tissues were treated ex vivo with lithium (5 and 50 mM) or valproate (10 and 100 mM, see Supporting information, Figure S2 for dosage calculations). A highly potent, non‐absorbable ASBT inhibitor, linerixibat, was used as a reference control. PCA of absorbed bile moieties (i.e., detected on the serosal side) revealed that valproate‐ and linerixibat‐treated samples clustered together and separately from the vehicle and lithium groups (Figure 6e). The analysis of individual bile compounds showed that valproate, similar to linerixibat, specifically inhibited the absorption of conjugated BA (Figure 6f and Supporting information, Table S4), indicating an inhibitory effect of the drug on the ASBT activity. We did not measure ASBT activity in chronically treated animals, but the valproate group displayed a stronger activation of Asbt expression (a 50% increase in Asbt protein abundance vs. a 30% increase in the lithium group), which might have been a compensatory response to a partial loss of ASBT activity. Lithium did not affect the absorption of conjugated BA under acute administration (Figure 6e,f and Supporting information, Table S4). Unconjugated BAs were overall not affected by either drug treatment, as these BAs can be passively absorbed and do not depend on the activity of the Asbt transporter.

In summary, these data indicate that lithium or valproate did not compromise the epithelial barrier function and did not cause major BA malabsorption in the intestine of chronically treated animals. On the contrary, the percentage of excreted bile was lower in treatment groups, indicating a net increase in bile uptake. Therefore, both drugs appear to directly interfere with FXR signalling in the small intestine, preventing activation of the Fxr/Fgf19 pathway.

## 4 DISCUSSION

### 4.1 Lithium and valproate disrupt FXR signalling in the liver and intestine

Here we demonstrate that chronic administration of lithium and valproate in rats induced a marked elevation of the BA pool in the systemic circulation, along with up‐regulation of hepatic Hmgcr, Cyp7a1 and Cyp27a1 proteins, which are the rate‐limiting enzymes in *de novo* BA synthesis, as well as an increase in the excreted faecal BA pool. These changes were most likely driven by the disruption of FXR signalling at both the hepatic and intestinal levels. In the intestine, we observed an impairment of Fxr‐mediated activation of the *Fgf19* gene (Figure 6d), which is a key suppressor of BA synthesis in rodents and plays a major role in the down‐regulation of Cyp7a1 in the liver (Kong et al., 2012). Additionally, the Fxr‐regulated adaptive response of bile transporters to the elevation of the BA pool, that is, down‐regulation of apical Asbt and basolateral Ostα/β transporters, was also desensitised in both treatment groups (Figure 6c). In the liver, despite a dramatic elevation of circulating BA levels, Fxr failed to suppress the expression of Cyp7a1, as well as Ntcp transporter, which mediates the BA uptake in the liver (Figure 4c,d).

Malfunctioning of Fxr signalling, particularly a deficit in the Fxr/Fgf19 negative feedback loop, results in overproduction of bile in the liver and an excessive load of BAs into the intestine. A similar phenotype, including an elevation of the BA pool and faecal BA excretion along with up‐regulation of hepatic *Cyp7a1* and *Hmgcr* expression, has been observed in animal models of disrupted Fxr/Fgf19 enterohepatic signalling, such as iFxr‐, Fgf15‐, Diet1‐deficient mice (dysregulation at the intestinal level) or Fgfr4‐ and β‐Klotho‐deficient mice (dysregulation at the hepatic level) (Cheng et al., 2018; Inagaki et al., 2005; Ito et al., 2005; Kong et al., 2012; Lee et al., 2018; Vergnes et al., 2013; Yu et al., 2013; Yu et al., 2000). Excessive bile load can have a negative impact on gut physiology, affecting microbiota composition, water and electrolyte secretion, motility and so forth (Hegyi et al., 2018).

### 4.2 Potential mechanisms of action

Despite showing many similarities in their effects, both drugs appear to have unique and selective impacts on Fxr signalling in both the liver and the intestine, indicating complex interactions with Fxr and its partners, as well as potentially different mechanisms of action. For example, in the liver, while the Fxr‐mediated down‐regulation of Cyp7a1 and Ntcp was lost, the up‐regulation of Bsep transporter, which mediates hepatic ‘efflux’ of BAs and is similarly controlled by activation of Fxr, was fully preserved in both treatment groups (Figure 4c,d). The up‐regulation of Shp, the key downstream target of Fxr, was observed in the Valproate but not in the Lithium group (Figure 4f). Furthermore, the analysis of the whole hepatic proteome showed that lithium had a relatively selective effect on hepatocytes, up‐regulating cholesterol and primary bile acid synthesis and activating fatty acid β‐oxidation (Supporting information, Figure S4). In contrast, chronic treatment with valproate induced a global restructuring of cellular bioenergetics in the liver, inhibiting the Krebs cycle and mitochondrial oxidative phosphorylation, with bile metabolic pathways being only a part of these changes (Supporting information, Figure S5). Interestingly, in the intestinal tissue, the effects of the drugs were much more subtle, implying multiple but mostly independent targets (Supporting information, Figures S7, S8).

In this study, we did not identify the exact mechanisms underlying the observed effects of drugs. The obstacles reside, first, in the broad range of targets the drugs can employ and, second, in the very complex nature of FXR signalling which involves numerous regulatory elements, including RXR, PXR, CAR, HNF4, MAFG and others (Chiang, 2015; Halilbasic et al., 2013; Perino et al., 2021). Based on existing knowledge, we can speculate that Li^+^ could have intervened in Fxr signalling by competing with Na^+^ and Mg^2+^ for interaction with numerous signalling/regulatory biomolecules that normally recognize these ions (Jakobsson et al., 2017). Li^+^ can modulate the activity of Na^+^ transporters and G‐protein‐coupled receptors. Upon entry into the cytosol, Li^+^ replaces the physiological cofactor Mg^2+^ and inhibits a series of metalloproteins such as G‐proteins, inositol phosphatases, adenylate cyclase, phospholipase C and protein kinases, all involved in cell signalling and therefore capable of modulating FXR function (Pasquali et al., 2010). Being of similar size, Li^+^ and Mg^2+^ also compete for binding with ATP and GTP. Depending on Li^+^ availability, the two ions can produce a variety of ATP–metal complexes that differentially affect metabolic and cell signalling pathways (Haimovich & Goldbourt, 2020). Moreover, Li^+^ possesses histone deacetylase inhibition ability (Wu et al., 2013); therefore, it could have reduced the transcription of Fxr targets, such as Fgf19, at the epigenetic level.

Similar to lithium, valproate can induce gene expression by causing changes in the state of histone acetylation across various tissues, including the liver. Moreover, valproate has been shown to act directly on DNA, changing its methylation patterns (Chateauvieux et al., 2010; Singh et al., 2021). Valproate was also shown to up‐regulate the activity of ERK (Zhang et al., 2014), which in turn can increase the stability of Shp (Miao et al., 2009) and hence contribute to the sustained activation of Cyp7a1. In this study, we have identified the inhibitory effect of valproate on Na^+^‐dependent Asbt transporter in the distal small intestine following acute treatment ex vivo (Figure 6e,f). Since Asbt transporter is responsible for the absorption of conjugated BAs into enterocytes (Dawson et al., 2003), a partial loss of its function could have potentially contributed to the loss of Fxr‐mediated regulation of Fgf19, Asbt and Osta/b (Figure 6c,d). However, this effect needs to be confirmed in a chronic treatment paradigm. Besides, the observed inhibition of Asbt activity could have been compensated by an up‐regulation of its protein abundance (Figure 6c). Finally, it should be noted that BA synthesis cannot be taken out of the context of glucose and lipid metabolism (Lefebvre et al., 2009). It is therefore possible that some changes in BA overproduction can be linked to the observed restructuring of cellular bioenergetics and lipid metabolism in the hepatic tissues (Supporting information, Figure S5). In conclusion, although the exact chain of events underlying the effects of lithium and valproate on the enterohepatic circulation of bile remains to be determined, we have discovered the FXR signalling pathway as their novel potential target.

### 4.3 Changes in the intestinal and hepatic absorption of bile in lithium‐ and valproate‐treated animals

In human and rodent studies, overproduction of bile commonly results in excessive loss of BAs in the colon, where they can induce watery diarrhoea by stimulating water and electrolyte secretion, as well as colonic motility (Farrugia & Arasaradnam, 2021; Lee et al., 2018). Interestingly, the overproduction of bile in our study was not associated with a massive elevation of excreted bile. While the plasma BA pool was elevated over 7‐fold, the faecal BA pool was increased only 1.6‐ to 1.8‐fold (Figures 1, 3). In other terms, the percentage of excreted bile (or faeces‐to‐plasma bile ratio) decreased from about 20% in the vehicle group to 3–4% in both treatment groups (Figure 3c), indicating an increase in BA uptake.

Several factors could have contributed to this effect. First, we observed a significant increase in the protein abundance of Asbt transporter, particularly in the Valproate group (Figure 6c), which suggests an up‐regulation of active uptake of conjugated bile moieties in the intestine, although in the case of valproate, the increase in protein expression under chronic administration of the drug was coupled with its inhibitory activity towards Asbt‐mediated transport of conjugated BAs under acute administration (Figure 6f). Second, both lithium‐ and valproate‐treated animals showed increased levels of glyco‐conjugated BAs (Figure 1c, Figures 2, 3). As discussed earlier, this most likely occurred as a compensatory response to deficits in taurine conjugation rates. Glyco‐conjugated BAs have substantially higher passive absorption rates compared to tauro‐conjugated forms (Schiff et al., 1972). Thus, an increased proportion of glyco‐BA would have resulted in increased efficacy of passive absorption of BAs and hence reduced BA loss in treated animals. Third, in both treatment groups, we observed a more substantial elevation of unconjugated compared to conjugated BAs: unconjugated BAs in plasma were increased 8‐fold, while conjugated BAs only 2‐fold (Figure 1c). Because unconjugated BAs have much higher passive absorption rates compared to conjugated forms (Schiff et al., 1972), these data also suggest an increased rate of passive bile reuptake.

It is worth noting that the unconjugated pool of BAs, although elevated, would bypass the FXR/FGF15/19 signalling in the intestine. Passive absorption of unconjugated BAs primarily occurs in the lower gut, following bacterial deconjugation, while the FXR/FGF15/19 axis is restricted to the distal small intestine. Although the presence of FXR/FGF15/19 signalling has also been shown in the colonic epithelial cells (Fallon et al., 2022), its physiological relevance is not known.

It should also be noted that we measured BAs in systemic blood, where – in contrast to the portal blood – the levels of BAs are determined not only by intestinal reabsorption but also by hepatic uptake. Thus, the observed elevation of the plasma BA pool might also suggest impairment of BA clearance by the liver. As mentioned earlier, we observed a larger elevation of unconjugated rather than conjugated BAs. A similar phenotype – a marked elevation of unconjugated BAs in plasma – has been described as a result of a deficiency in the activity of Na^+^‐independent organic anion‐transporting polypeptide (OATP) transporters in the liver (Csanaky et al., 2011; Slijepcevic et al., 2015). Unlike Na^+^‐dependant NTCP transporters, OATPs carry the vast majority of unconjugated BAs from the blood into the liver. This observation is quite intriguing and difficult to explain. Indeed, based on its chemical properties, lithium is more likely to affect the activity of Na^+^‐dependant transporters (Jakobsson et al., 2017). Valproate was shown to have an inhibitory effect on Na^+^‐dependent conductance (VanDongen et al., 1986). In our study, valproate showed inhibitory activity towards the intestinal uptake of conjugated BAs mediated by Asbt transporter (Figure 6f), which is sodium‐dependent and belongs to the same family of SLC10 transporters as NTCP. We must acknowledge, though, that we did not measure the activity of hepatic transporters. This research is warranted in future studies to better understand the impact of lithium and valproate on liver transporters. This might be of particular interest because OATP transporters are also involved in the hepatic update of drugs and xenobiotics (Jetter & Kullak‐Ublick, 2020).

### 4.4 Disruption of FXR signalling and enterohepatic circulation of bile: potential consequences in and beyond the intestine

There is limited information on the effects of lithium and valproate on gastrointestinal physiology. Both drugs exhibit protective effects on the intestine in disease states. For example, preclinical studies have shown that lithium exerts protective and anti‐inflammatory properties in animal models of colitis and gastric damage (Daneshmand et al., 2009; Daneshmand et al., 2011; Huang et al., 2022; Nejadkey et al., 2006). Lithium therapy also ameliorated intestinal mucosal injury in patients with severe graft‐versus‐host disease (Steinbach et al., 2017). Similarly, valproate decreased tissue damage and suppressed inflammation in experimental colitis and in cyclophosphamide‐induced cytotoxicity in mouse colon (Glauben et al., 2006; Khan & Jena, 2014). In line with these findings, our studies did not observe intestinal inflammation or damage to intestinal barrier function with acute and chronic lithium or valproate administration (Supporting information, Figure S9B–D; Cussotto et al., 2019). Proposed mechanisms behind these effects include modulation of nitric oxide signalling (Nejadkey et al., 2006; Shamshiri et al., 2009), activity of ATP‐sensitive potassium channels (Daneshmand et al., 2011) and production of bacteria‐derived short‐chain fatty acids (Huang et al., 2022).

Our findings show that both lithium and valproate suppress bile‐activated Fxr/Fgf19 negative feedback signalling in the intestine and Fxr/Shp signalling in the liver, inducing excessive BA synthesis. Overproduction of bile commonly results in the spillage of BAs in the colon, inducing water and electrolyte secretion, stimulating motility and causing diarrhoea (Farrugia & Arasaradnam, 2021). Bile‐associated diarrhoea (BAD) can result from either overproduction of bile in the liver (type 2, idiopathic or primary BAD) or malabsorption of bile in dysfunctional small intestine (types 1 and 3 BAD, e.g., in Crohn's disease, celiac disease, ileal resection) (Camilleri, 2015; Camilleri, 2021). Intriguingly, similar pathophysiological changes, such as impairment of enteric FGF19 signalling and overproduction of bile in the liver, have been described as a cause of primary BAD in humans (Pattni et al., 2013; Walters, 2014).

Clinical studies report diarrhoea as a common reason for discontinuation of both lithium and valproate (Jahromi et al., 2011; Öhlund et al., 2018); however, the underlying mechanisms have not been described. Our findings suggest that both drugs can induce primary BAD via suppression of FXR/FGF19 negative feedback signalling and subsequent activation of BA synthesis in the liver. The prevalence of BAD in patients under lithium and valproate therapy has not been looked at so far. Nonetheless, our findings provide a strong rationale to investigate BAD as a complication of these drugs in human studies. BAD can be diagnosed by measuring ^75^SeHCAT (selenium‐75 homocholic acid taurine) retention values or through combined measures of fasting C4 (7α‐hydroxy‐4‐cholesten‐3‐one, hepatic synthesis) and FGF19 levels in the blood (Pattni et al., 2013; Walters, 2014). BAD is generally well managed with bile acid sequestrants (Farrugia & Arasaradnam, 2021; Walters & Pattni, 2010). Screening for BAD in patients taking lithium and valproate with diarrhoea can potentially improve patient adherence to therapy.

In our study, we could not correlate changes in BA metabolism with gastrointestinal motility. We used biological samples generated in our previous work, which focused on the impact of chronic administration of psychotropic medications on gut microbiota composition (Cussotto et al., 2019) and did not include gastrointestinal motility readouts in the design. Additionally, BA metabolism in rats differs from humans in various aspects, including bile synthesis (Thakare et al., 2018a; Thakare et al., 2018b) and storage (absence of gall bladder), amongst others. Despite these limitations, we believe our findings will inspire further studies to explore the proposed connection between changes in BA metabolism and diarrhoea in patients undergoing lithium and valproate therapy.

Outside the intestine, BAs and FXR signalling are crucial components of lipid and glucose metabolic pathways. BAs are synthesised from cholesterol, and increased BA production can be interpreted as a metabolic response to cholesterol excess. However, we did not find evidence supporting this speculation. First, we did not see any evidence of activated cholesterol uptake in the intestine. When we looked at proteins involved in intestinal cholesterol absorption, we did not observe changes in the expression of Npc1l1 (apical uptake of cholesterol), Acat2 (transcellular trafficking) or Ldlr (basolateral transport) (Supporting information, Figure S11). Second, the plasma cholesterol levels were either unchanged or decreased in treatment groups (Figure 1e). Hence, it is more likely that it is the activation of BA synthesis that drives the observed up‐regulation of Hmgcr expression (Figure 4c) and cholesterol synthesis. Since cholesterol and triglycerides (TG) share acetyl‐CoA as a precursor, increased cholesterol synthesis might have contributed to the reduction in systemic TG levels, white fat deposition and body weight gain observed in both treatment groups (Figure 1e and Supporting information, Figure S10). These findings contradict clinical observations as these medications typically induce weight gain (Ackerman & Nolan, 1998). We also hypothesized the opposite, that is, a decrease in body weight causing an increase in circulating BAs. However, studies in humans have shown a positive correlation between body weight gain and total BA levels (Alemán et al., 2018; Prinz et al., 2015).

Another interesting effect of lithium and valproate is the reduction of tauro‐conjugated BA and hepatic taurine levels (Figure 2c,d). Taurine is an organic compound widely distributed in animal tissues with many fundamental biological roles, including antioxidation (Jong et al., 2012; Yildirim & Kilic, 2011), osmoregulation (Schaffer et al., 2000), cardiovascular function (Schaffer et al., 2010; Xu et al., 2008) and brain function (Wu & Prentice, 2010). Taurine can cross the blood–brain barrier (Salimäki et al., 2003; Tsuji & Tamai, 1996; Urquhart et al., 1974) and produce a central anxiolytic effect, probably through activation of the glycine receptor (Kong et al., 2006; Zhang & Kim, 2007). In animals treated with lithium and valproate, taurine was depleted not only in the liver but also in circulation (Figure 1d), potentially causing a shortage in the central nervous system. Some evidence exists on how the mood stabilizers lithium and valproate can influence brain levels of taurine (Anyanwu & Harding, 1993; O'Donnell et al., 2003; Pettegrew et al., 2001). We observed a significant down‐regulation of the hepatic taurine transporter *TauT* gene in both treatment groups (Supporting information, Figure S6C) and here we hypothesize a mechanism implying the inhibitory effect of the drugs on taurine transport into the central nervous system via TauT transporter, which is also expressed in the brain (Pow et al., 2002). Future studies should investigate the relationship between psychotropic drug action and central taurine levels.

### 4.5 Concluding remarks

The present study demonstrates that lithium and valproate disrupt bile‐activated FXR signalling at both hepatic and intestinal levels, resulting in a substantial increase in BA production in rats. These findings offer new insights into the targets of lithium and valproate within and beyond the gastrointestinal tract. Furthermore, given the striking resemblance of the observed changes to the pathophysiology of primary BAD, this research provides a rationale for investigating the implication of BAD in patients receiving lithium and valproate in clinical studies.

## AUTHOR CONTRIBUTIONS

Sofia Cussotto, Anna V. Golubeva, Susan A. Joyce, Timothy G. Dinan and John F. Cryan designed the experiment. Sofia Cussotto conducted the preclinical experiment and biochemical analyses. Alvaro Lopez Gallardo performed the bile acid extraction, quantification, data analysis and interpretation. Anna V. Golubeva, Caitriona Scaife and Jane A. English performed the proteomic analysis and assisted with the intestinal permeability experiment and data interpretation. Susan A. Joyce, Gerard M. Moloney and Alexander V. Zhdanov assisted with the formulation of working hypotheses. Thomaz F.S. Bastiaanssen performed the analysis of 16S sequencing and assisted with the analysis of proteomic data. Sofia Cussotto and Anna V. Golubeva wrote the initial draft of the manuscript. All authors critically revised the manuscript. All authors have read and approved the final version of this manuscript and agree to be accountable for all aspects of the work in ensuring that questions related to the accuracy or integrity of any part of the work are appropriately investigated and resolved. All persons designated as authors qualify for authorship, and all those who qualify for authorship are listed.

## CONFLICT OF INTEREST

J.F.C. has been an invited speaker at conferences organized by Bromotech and Nestle and has received research funding from the Saks Kavanaugh Foundation, Nutricia, Dupont/IFF and Nestle. This support neither influenced nor constrained the contents of this article. All other authors report no financial interests or potential conflicts of interest.

## Supporting information



Supplementary Methods.

Supplemental Figures S1–S11.

Supplementary Tables S1–S4.

Table S5. List of SYBR Green probes used in the study.

Proteome data in the liver tissue following chronic administration of lithium and valproate.

Proteome data in the ileum tissue following chronic administration of lithium and valproate.

## Data Availability

The data used to generate main and supplementary figures are available in online supporting information (Tables S1, S2, S3, S4, S6 and S7). All original code has been deposited at GitHub and is publicly available (https://github.com/thomazbastiaanssen/Tjazi). STRING functional enrichment analysis is publicly available at https://string‐db.org/.
