# Supplementary material for: The mood stabilizers lithium and valproate disrupt hepatic and intestinal farnesoid X receptor signalling and increase bile synthesis in the rat
Source: Exp Physiol. 2025 Mar 28;110(9):1233–53. doi: 10.1113/EP092451 (PMC12400835; doi:10.1113/EP092451)
Supplement: Supplementary file 1 — Supplementary Methods. [file EPH-110-1233-s002.docx]

**SUPPLEMENTARY METHODS**

**LC/MS analysis of bile acids in plasma, faeces, liver of chronically treated animals, and Ussing chamber aliquots**

***Extraction*** Bile acids were extracted according to Joyce et al. (Joyce, MacSharry et al. 2014). For the faeces, 80 mg of faecal material was added to dynabeads (Roche) with 300 μl of ice-cold 50% methanol containing deuterated internal standards of both Cholic acid and Chenodeoxycholic acid, then subjected to five 30-second rounds of extraction in a dyna-lyser machine (Roche) at 6000 rpm. For plasma extractions, 100 μl was mixed with 300 μl of ice-cold 50% methanol also containing deuterated internal standards. Each mixture was vortexed and then centrifuged for 10 mins at 10000 g and the supernatant was transferred to a fresh tube. Ice cold acetonitrile (ACN) with formic acid measuring 2 ml was added to each tube, vortexed and agitated at room temperature for 1 hour. Samples were centrifuged again to pellet the debris and the supernatant was added to fresh tubes containing 1 ml ice-cold 100 % ACN. The samples were vortexed and dried under a vacuum at +4°C. The dried extracted acids were re-suspended in 150 μl of ice-cold 50 % methanol.

***Chemicals*** Standard Conjugated bile salts and free bile acids were purchased from Sigma Aldrich and Steraloids, Inc. (Newport, Rhode Island). HPLC-grade methanol, acetonitrile, water, ammonium acetate, ammonium formate, ammonium hydroxide, formic acid, and acetic acid and water were obtained from Fisher Scientific (Fair Lawn, NJ). Deuterated cholic acid (D-2452) and deuterated chenodeoxycholic acid (D-2772) were purchased from CDN Isotopes Inc. Standards were constructed as 1mg/ml stock solutions of individual sulfated BAs were prepared in water:MeOH (1:1). They were subsequently combined to a final volume of 1.0 ml in water to give a stock concentration of 40 mg/ml for each. Subsequent dilutions were made to which the same volume of deuterated standards was added. Fatty acids were treated similarly but were resuspended in 100% methanol. These standards were utilized to create standard curves for each analyte examined.

***Ultra Performance Liquid Chromatography Tandem Mass Spectrometry*** UPLC-MS was performed using a modified method by Joyce et al. (Joyce, MacSharry et al. 2014). Briefly, 5μL from each sample was injected onto a C18 Acquity column (Waters Corp.). Each sample was run in triplicate. Extracts were eluted using a 25-min gradient of 100 % A to 100 % B (A, water, 0.1 % formic acid; B, acetonitrile, 0.1 % formic acid) at a flow rate of 500 μL/min and column temperature of 40°C. Samples were analyzed using an Acquity system (Waters Ltd.) coupled online to an LCT Premier mass spectrometer (Waters MS Technologies, Ltd.) in negative electrospray mode with a scan range of 50–1,000 m/z. Bile acids ionize strongly in negative mode, producing a prominent [M-H]− ion. Capillary voltage was 2.4 Kv, sample cone was 35 V, desolvation temperature was 350°C, source temperature was 120°C, and desolvation gas flow was 900 L/h. Analysis was performed using Waters software Targetlynx for exact quantification against a standard curve for each analyte and Markerlynx for non-biased PCA analysis.

**Proteomic analysis in the liver and ileum tissues of chronically treated animals.**

***Protein extraction*.** Total protein were extracted, purified and prepared for mass spectrometry (MS) using iST Mammalian Tissue Sample Preparation Kit (PREOMICS, Germany) according to the manufacturer’s protocol with minor modifications. Specifically, 10 – 20 mg of tissue were homogenised in 400 μL of LYSE buffer using Vibra Cell Sonicator (Sonics #VCX 130PB) at 40% of power, 8-10 pulses of 5 sec each, with 30-sec resting interval between pulses. 100 μg of protein were further digested and purified; the peptides were re-suspended in 100 μl of LC-LOAD buffer.

***LC-MS/MS analysis.*** For LC-MS/MS analysis, samples were loaded onto individual EvoTips and run on a timsTOF Pro mass spectrometer (Bruker Daltonics, Bremen, Germany) coupled to the EvoSep One system (EvoSep BioSystems, Odense, Denmark). The peptides were separated on a reversed-phase C18 Endurance column (15cm x 150μm ID, C18, 1.9 μm) using the preset 30 SPD method. Mobile phases were 0.1% (v/v) formic acid in water (phase A) and 0.1% (v/v) formic acid in acetonitrile (phase B). The peptides were separated by an increasing gradient of mobile phase B for 44 minutes using a flow rate of 0.5 μL/min. For Data Dependent Acquisition (DDA) the timsTOF Pro mass spectrometer was operated in positive ion polarity with TIMS (Trapped Ion Mobility Spectrometry) and PASEF (Parallel Accumulation Serial Fragmentation) modes enabled. The accumulation and ramp times for the TIMS were both set to 100 ms, with an ion mobility (1/k0) range from 0.6 to 1.6 Vs/cm. Spectra were recorded in the mass range from 100 to 1,700 m/z. The precursor (MS) Intensity Threshold was set to 2,500 and the precursor Target Intensity set to 20,000. Each PASEF cycle consisted of one MS ramp for precursor detection followed by 10 PASEF MS/MS ramps, with a total cycle time of 1.17 s. For Data Independent Acquisition (DIA) on the timsTOF Pro, a diaPASEF scheme consisting of 46 precursor isolation windows of 25 m/z width, covering a mass range of 300 – 1450 m/z and an ion mobility range of 0.6 to 1.6 Vs/cm, was created using the Bruker timsControl interface (2.0.53).

***Protein Identification Pipeline***. For the analysis of DDA and DIA (diaPASEF) data the FragPipe computational proteomics platform (version 19.1) was used with the DIA_SpecLib_Quant workflow selected. In this workflow DDA data was first searched using MSFragger (version 3.7) (Kong, Leprevost et al. 2017), (Teo, Polasky et al. 2021) against the Uniprot reference proteome for Rattus norvegicus (UP000002494_10116; downloaded 08/03/2023) to which decoy sequences and common contaminants were appended. MSFragger features were combined with MSBooster (version 1.1.11) generated deep learning based features, and PSMs (Peptide Spectrum Matches) were rescored with Percolator (version 3.05.0) (Käll, Canterbury et al. 2007). Proteins were inferred with ProteinProphet (version 4.8.0) (Nesvizhskii, Keller et al. 2003). Next, a spectral library created from the DDA search results with the python package EasyPQP (version 0.1.36) was used as input for DIA-NN (version 1.8.2 beta 8) for searching and quantitation of diaPASEF data (da Veiga Leprevost, Haynes et al. 2020, Demichev, Szyrwiel et al. 2022). All default settings for the individual modules within this workflow were accepted.

***Statistical Analysis.*** The diann-output.pg_matrix file containing normalized intensities for protein groups was used for subsequent statistical analysis in Rstudio GUI (version 2022.2.2.485). First, identified proteins were filtered to include proteins with >70% of valid values in at least one experimental group. Next, the intensity values were clr transformed using the Tjazi library (Bastiaanssen, Quinn et al. 2023, Bastiaanssen, Quinn et al. 2023). Zero count values were imputed using the ‘const’ method, taking 2/3 of the lowest non-zero sample value, as described by Lubbe and colleagues (Lubbe, Filzmoser et al. 2021). Beta diversity was computed in terms of Aitchison distance, or Euclidean distance between clr-transformed data; principal component analysis (PCA) was used for visualization. The differences in protein expression levels between groups were analyzed using protein-wise generalized linear models (GLMs). Dunnett’s test with the Vehicle group as a control category was used for post hoc to account for multiple comparisons. To correct for multiple testing, the Benjamini-Hochberg post hoc procedure was performed with a false discovery rate (FDR) q-value of 0.05 as a cut-off (Benjamini and Hochberg 1995). Differentially abundant proteins with estimated effect size > 0.5 and < -0.5 on the log2-fold scale were selected for STRING pathway analysis <https://string-db.org/> (Szklarczyk, Gable et al. 2019). Plotting was handled using ggplot2. All R scripts are available online at <https://github.com/thomazbastiaanssen/Tjazi> (Bastiaanssen, Quinn et al. 2023, Bastiaanssen, Quinn et al. 2023).

**References**

1. Bastiaanssen, T. F. S., T. P. Quinn and A. Loughman (2023). "Bugs as features (part 1): concepts and foundations for the compositional data analysis of the microbiome–gut–brain axis." Nature Mental Health **1**(12): 930-938.
2. Bastiaanssen, T. F. S., T. P. Quinn and A. Loughman (2023). "Bugs as features (part 2): a perspective on enriching microbiome–gut–brain axis analyses." Nature Mental Health **1**(12): 939-949.
3. Benjamini, Y. and Y. Hochberg (1995). "Controlling the false discovery rate: a practical and powerful approach to multiple testing." Journal of the Royal statistical society: series B (Methodological) **57**(1): 289-300.
4. da Veiga Leprevost, F., S. E. Haynes, D. M. Avtonomov, H. Y. Chang, A. K. Shanmugam, D. Mellacheruvu, A. T. Kong and A. I. Nesvizhskii (2020). "Philosopher: a versatile toolkit for shotgun proteomics data analysis." Nat Methods **17**(9): 869-870.
5. Demichev, V., L. Szyrwiel, F. Yu, G. C. Teo, G. Rosenberger, A. Niewienda, D. Ludwig, J. Decker, S. Kaspar-Schoenefeld, K. S. Lilley, M. Mulleder, A. I. Nesvizhskii and M. Ralser (2022). "dia-PASEF data analysis using FragPipe and DIA-NN for deep proteomics of low sample amounts." Nat Commun **13**(1): 3944.
6. Joyce, S. A., J. MacSharry, P. G. Casey, M. Kinsella, E. F. Murphy, F. Shanahan, C. Hill and C. G. M. Gahan (2014). "Regulation of host weight gain and lipid metabolism by bacterial bile acid modification in the gut." Proceedings of the National Academy of Sciences of the United States of America **111**(20): 7421-7426.
7. Käll, L., J. D. Canterbury, J. Weston, W. S. Noble and M. J. MacCoss (2007). "Semi-supervised learning for peptide identification from shotgun proteomics datasets." Nature Methods **4**(11): 923-925.
8. Kong, A. T., F. V. Leprevost, D. M. Avtonomov, D. Mellacheruvu and A. I. Nesvizhskii (2017). "MSFragger: ultrafast and comprehensive peptide identification in mass spectrometry–based proteomics." Nature methods **14**(5): 513-520.
9. Lubbe, S., P. Filzmoser and M. Templ (2021). "Comparison of zero replacement strategies for compositional data with large numbers of zeros." Chemometrics and Intelligent Laboratory Systems **210**: 104248.
10. Nesvizhskii, A. I., A. Keller, E. Kolker and R. Aebersold (2003). "A statistical model for identifying proteins by tandem mass spectrometry." Anal Chem **75**(17): 4646-4658.
11. Szklarczyk, D., A. L. Gable, D. Lyon, A. Junge, S. Wyder, J. Huerta-Cepas, M. Simonovic, N. T. Doncheva, J. H. Morris, P. Bork, L. J. Jensen and C. V. Mering (2019). "STRING v11: protein-protein association networks with increased coverage, supporting functional discovery in genome-wide experimental datasets." Nucleic Acids Res **47**(D1): D607-D613.
12. Teo, G. C., D. A. Polasky, F. Yu and A. I. Nesvizhskii (2021). "Fast Deisotoping Algorithm and Its Implementation in the MSFragger Search Engine." J Proteome Res **20**(1): 498-505.
