# Supplementary material for: The mood stabilizers lithium and valproate disrupt hepatic and intestinal farnesoid X receptor signalling and increase bile synthesis in the rat
Source: Exp Physiol. 2025 Mar 28;110(9):1233–53. doi: 10.1113/EP092451 (PMC12400835; doi:10.1113/EP092451)
Supplement: Supplementary file 2 — Supplemental Figures S1–S11. [file EPH-110-1233-s003.docx]

**SUPPLEMENTAL FIGURES**

**Figure S1. Experimental details of *in vivo* and *ex vivo* experiments.** For panel A, the predicted function of the gut microbiota was analyzed using the 16S sequencing data generated in a previous study [Cussotto, S., et al., Differential effects of psychotropic drugs on microbiome composition and gastrointestinal function. Psychopharmacology (Berl), 2019. 236(5): p. 1671-1685]. *Abbreviations:* ALT - Alanine transaminase, AST - aspartate transaminase, ASBT - apical sodium-dependent bile acid transporter, BA - bile acid, BW - body weight*,* Fgf19 - fibroblast growth factor-19, FITC - fluorescein isothiocyanate, *Isc* - short-circuit current, SD - Sprague-Dawley, TEER - transepithelial electrical resistance.

**
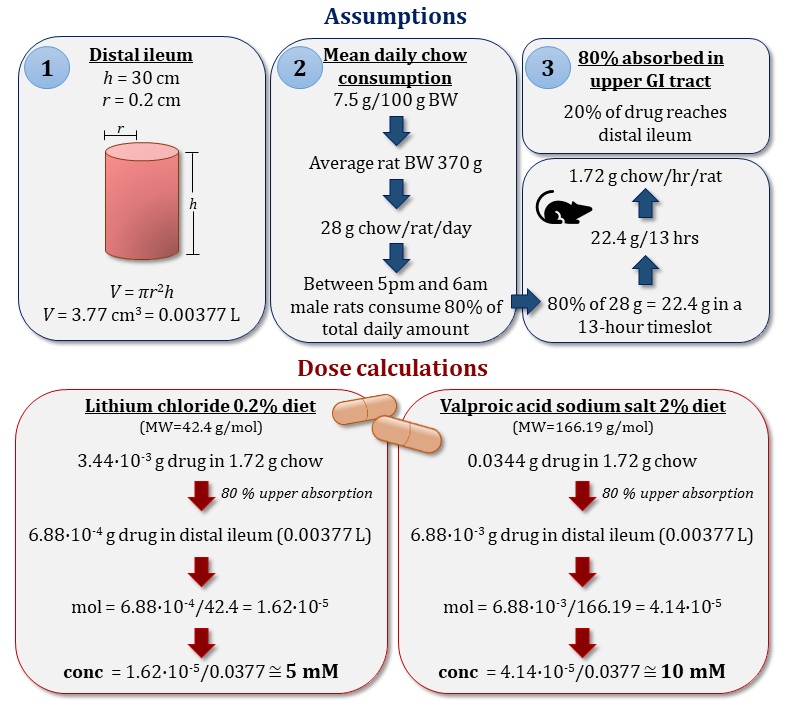
**

**Figure S2. Dose calculation of lithium and valproate for the *ex vivo* assessment of intestinal permeability and BA absorption.** *Abbreviations:* BW - body weight, MW - molecular weight. Reference for assumption 1: doi.org/10.1203/00006450-200208000-00023. Reference for assumption 2: PMID 8624748. References for assumption 3: doi.org/10.1007/978-1-4684-2022-7_4 and doi.org/10.2165/00003088-198005010-00002.


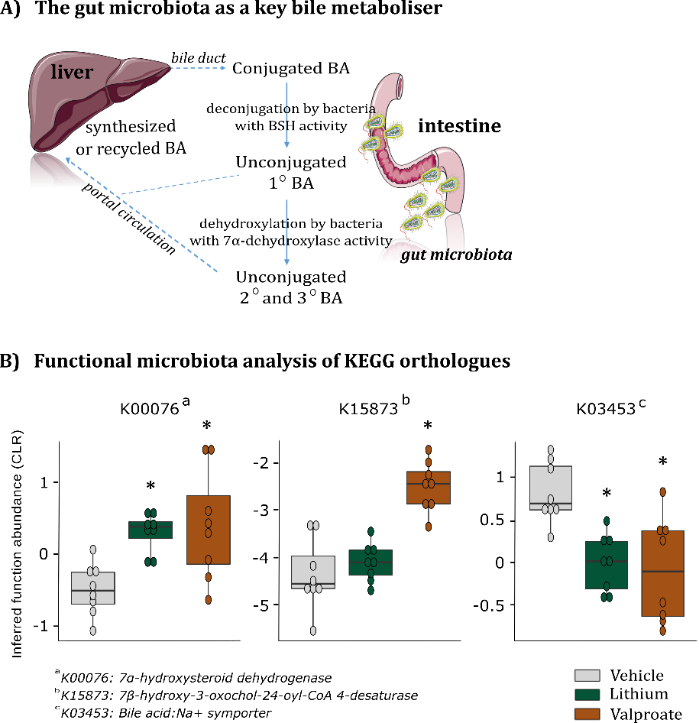


**Figure S3. Lithium and valproate alter the abundance of molecular functions implicated in primary-to-secondary bile metabolism and bile transport**. (A) Schematic of bile metabolism by the gut microbiota. (B) Predicted KEGG orthologue functional assay. Lithium increased the inferred function abundance of K00076 and decreased the inferred function abundance of K03453. Valproate increased both K00076 and K15873, and decreased K03453. Data are expressed as median, IQR, and min-to-max values. *p<0.05 (n=8/group). Data were analysed using the Kruskal-Wallis test followed by Mann-Whitney U-test.


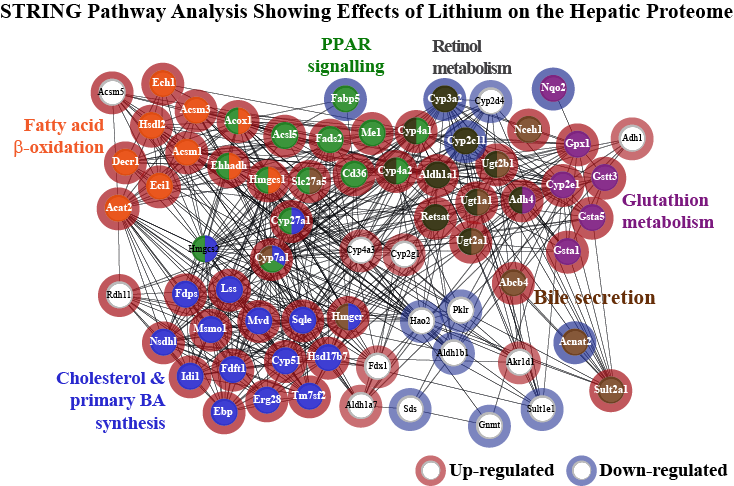


**Figure S4**. **STRING pathway analysis showing effects of lithium on the hepatic proteome.** Only significantly affected (FDR adjusted p<0.05) proteins with an effect size >│0.5│on the log2-fold scale were included in the analysis. See **Table S6** for statistical details. Chronic treatment with lithium significantly changed the expression of 547 proteins, with 229 proteins being up-regulated and 318 being down-regulated by lithium. We further chose the proteins with the strongest effect size (EE >0.5 or <-0.5 on the log2-fold scale) which narrowed the list to 87 up-regulated and 45 down-regulated proteins. For functional enrichment analysis, these proteins were uploaded to STRING (https://string-db.org/). The strongest significantly implicated (FDR adjusted p<0.05) protein pathways were colour-coded in the generated protein interaction network. The analysis revealed that chronic administration of lithium had the largest impact on cholesterol and primary bile acid synthesis, up-regulating 17 of 37 associated proteins (BLUE). This was accompanied by a concomitant activation of fatty acid beta-oxidation (ORANGE). Other pathways affected by lithium included: *PPAR signaling pathway* (GREEN); *Retinol metabolism* (DARK GREY); *Bile secretion* (BROWN); *Glutathione metabolism, and detoxification of reactive oxygen species* (PURPLE). Red and blue halos indicate significantly increased and decreased protein content, respectively.

| **Colour** | **Pathway source** | **Pathway ID** | **Pathway Name** | **Count in Network** | **Strength** | **P value after FDR** |
| --- | --- | --- | --- | --- | --- | --- |
| **BLUE** | STRING | CL:10714 | *Cholesterol biosynthesis, and primary bile acid biosynthesis* | 17 of 37 | 1.89 | 4.84E-22 |
| **ORANGE** | STRING | CL:9439 | *Fatty acid beta-oxidation, and butanoate metabolism* | 10 of 49 | 1.53 | 5.24E-10 |
| **GREEN** | KEGG | rno03320 | *PPAR signaling pathway* | 14 of 77 | 1.48 | 4.12E-14 |
| **DARK GREY** | KEGG | rno00830 | *Retinol metabolism* | 10 of 62 | 1.43 | 7.29E-10 |
| **BROWN** | KEGG | rno04976 | *Bile secretion* | 10 of 78 | 1.33 | 4.95E-09 |
| **PURPLE** | STRING | CL:10191 | *Glutathione metabolism, and detoxification of reactive oxygen species* | 7 of 92 | 1.1 | 0.00036 |


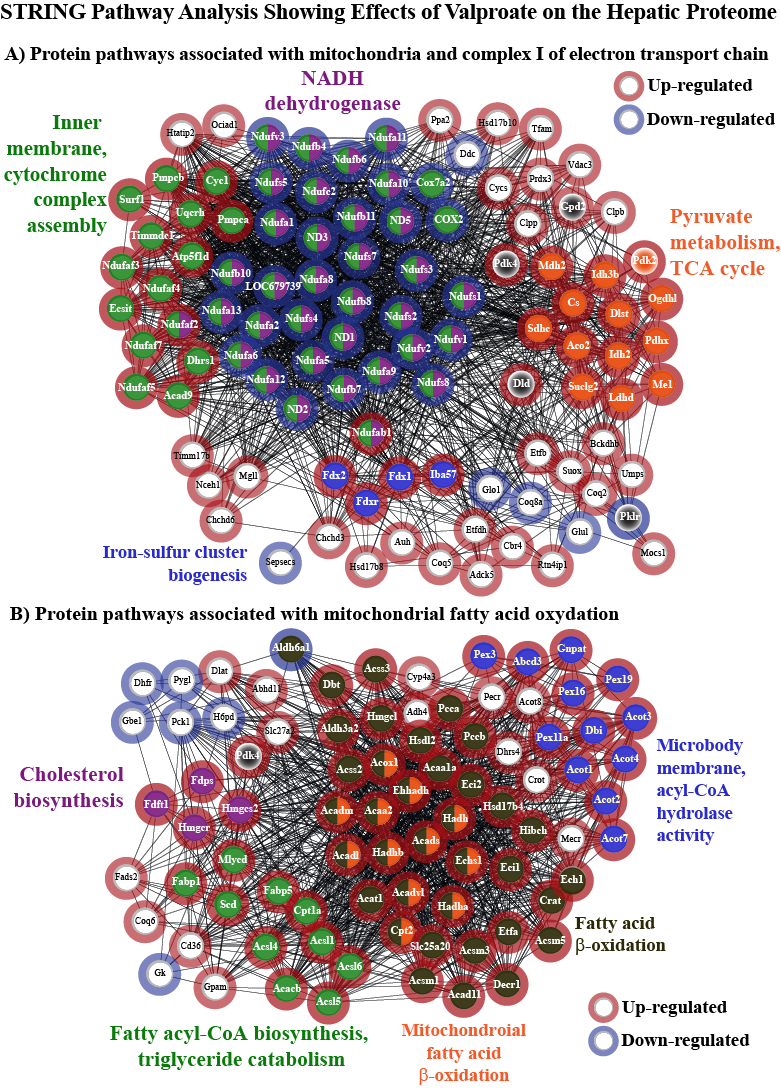


**Figure S5**. **STRING pathway analysis showing effects of valproate on the hepatic proteome.** Only significantly affected (FDR adjusted p<0.05) proteins with an effect size >│0.5│on the log2-fold scale were included in the analysis. See **Table S6** for statistical details. Chronic treatment with valproate had a very strong effect on the liver proteome, with 1838 proteins being significantly affected (785 up-regulated and 1053 down-regulated proteins). After filtering for the effect size, we reduced the list to 477 up-regulated and 392 down-regulated proteins. For functional enrichment analysis, these proteins were uploaded to STRING (https://string-db.org/) to reveal the implicated pathways. The most strongly affected pathways of interest were colour-coded on the generated interaction networks **(A and B)**. Proteins that are individually discussed below are shown as gradient circles. Red and blue halos indicate significantly increased and decreased protein content, respectively.

1. Protein pathways associated with mitochondria and complex I of electron transport chain.

| **Colour** | **Pathway source** | **Pathway ID** | **Pathway Name** | **Count in Network** | **Strength** | **P value after FDR** |
| --- | --- | --- | --- | --- | --- | --- |
| **PURPLE** | STRING | CL:12058 | *NADH dehydrogenase complex* | 34 of 52 | 2.15 | 3.84E-56 |
| **GREEN** | STRING | CL:12032 | *Inner mitochondrial membrane protein complex, and cytochrome complex assembly* | 50 of 186 | 1.77 | 3.83E-69 |
| **BLUE** | STRING | CL:11828 | *Mitochondrial iron-sulfur cluster biogenesis, and MOSC, N-terminal beta barrel* | 4 of 13 | 1.83 | 0.00013 |
| **ORANGE** | STRING | CL:9207 | *Pyruvate metabolism and citric acid (TCA) cycle, and oxaloacetate metabolic process* | 13 of 48 | 1.77 | 3.05E-16 |

1. Protein pathways associated with mitochondrial fatty acid oxidation.

| **Colour** | **Pathway source** | **Pathway ID** | **Pathway Name** | **Count in Network** | **Strength** | **P value after FDR** |
| --- | --- | --- | --- | --- | --- | --- |
| **ORANGE** | STRING | CL:9448 | *Mitochondrial fatty acid beta-oxidation of saturated fatty acids, and acyl-coenzyme A oxidase N-terminal* | 12 of 12 | 2.43 | 3.00E-21 |
| **DARK GREY** | STRING | CL:9434 | *Mixed, including fatty acid beta-oxidation, and valine, leucine and isoleucine degradation* | 36 of 103 | 1.97 | 2.66E-55 |
| **GREEN** | STRING | CL:10929 | *Fatty acyl-CoA biosynthesis, and triglyceride catabolism* | 10 of 32 | 1.92 | 1.26E-13 |
| **PURPLE** | STRING | CL:10718 | *Cholesterol biosynthesis* | 4 of 20 | 1.73 | 0.00029 |
| **BLUE** | STRING | CL:11375 | *Microbody membrane, and acyl-CoA hydrolase activity* | 12 of 65 | 1.69 | 3.24E-14 |

STRING analysis revealed that chronic administration of valproate primarily affected mitochondria. Out of 39 protein clusters affected by valproate administration in the Local Network Cluster (STRING), 33 clusters, or 85% were associated with mitochondria. A remarkable increase was seen in the levels of proteins implicated in mitochondrial biogenesis, membrane remodelling, and crista development (e.g., Ndufaf2, Ndufaf4, Ndufaf5, Ndufaf7, Surf1, Iba57, Acad9), protein import and processing (e.g., Timmdc1, Timm17b, Pmpca, Pmpcb), as well as mitochondrial metabolism (e.g., Uqcrh, Cycs, Mdh2, Aco2, Idh2, Ldhd, Atp5f1d). The triggering factor behind these changes was most likely the inhibitory effect of valproate on the Krebs cycle. Valproate and its intermediates can form conjugates with coenzyme A (CoA). Valproyl-CoA has been shown to directly inhibit the activity of the hepatic dihydrolipoamide dehydrogenase (DLD), which is an essential component of the pyruvate dehydrogenase (PDH) and 2-oxoglutarate (2-OG) dehydrogenase (OGDH) complexes (Kudin, Mawasi et al. 2017). These findings agree with previously reported inhibitory effects of valproate on pyruvate- and 2-OG-driven oxidative phosphorylation in hepatotoxicity models (Silva, Ruiter et al. 1997, Luis, Ruiter et al. 2007). Persistent suppression of PHD and OGDH - critical enzymes in the Krebs cycle - would have resulted in a substantial depletion of NADH and FADH_2_ supply to the electron transport chain. This would have also pushed mitochondria towards major metabolic rewiring, i.e., an up-regulation of proteins involved in mitochondrial respiration, directed to compensate for the inhibition of pyruvate and 2-OG oxidation. Supporting this hypothesis, we observed an increase in Dld expression (**A**, grey), as well as a concomitant elevation in the levels of other components within the PDH and OGDH complexes (**A**, Pdha1, Dlat, Dlst, Ogdh, green and orange).

On the other hand, we noted changes suggesting that the observed restructuring of mitochondria in valproate-treated livers, however extensive it was, might not be very efficient. Firstly, although we did not measure pyruvate levels directly, we saw a reduction in the expression of pyruvate kinase Pklr (**A**, grey), an enzyme responsible for the transphosphorylation of phosphoenolpyruvate into pyruvate and ATP. This step is the final and rate-limiting process in glycolysis that delivers pyruvate to the PDH complex. These data suggest decreased pyruvate availability in valproate-treated tissues. Secondly, a significant increase was observed in the levels of PDH kinase 2 (Pdk2, **A**, orange). This kinase is regarded as an important player in rapid metabolic adaptability within the liver via the inhibition of the PDH complex (Woolbright and Harris 2021), which conducts the irreversible oxidation of pyruvate and generates acetyl-CoA, thus linking glycolysis and the Krebs cycle. Likewise, the expression of PDK4, which typically rises concurrently with increasing blood concentrations of fatty acids (FA) and inhibits PDH (Pettersen, Tusubira et al. 2019), was also elevated (**A** and **B**, grey). This suggests that the probability of PDH inhibition through the phosphorylation by PDK2 and PDK4 is very high in the liver tissues upon valproate administration. In these conditions, the rates of glycolysis and pyruvate oxidation by PDH complex are expected to synchronously decrease, to give way to alternative pathways involved in the ATP production, such as fatty acid beta-oxidation (FAO). In concordance, the elevation of PDK4 levels is typically recognized as indicative of a metabolic transition from glycolysis to FAO (Pettersen, Tusubira et al. 2019).

Indeed, our data showed the up-regulation of a wide array of proteins linked to mitochondrial FAO in valproate-treated animals, suggesting increased activity of this pathway to compensate for the deficiency in pyruvate oxidation (**B**, orange). Unlike pyruvate oxidation, which generates solely NADH molecules in the Krebs cycle, FAO supplies the electron transport chain with both NADH and FADH_2_. The glycerol-3-phosphate shuttle also contributes to the mitochondrial FADH_2_ pool, and we noted a substantial elevation in the quantities of mitochondrial glycerol-3-phosphate dehydrogenase (GPD2, **A**, grey) the pivotal enzyme within this shuttle pathway. These changes would have increased the mitochondrial FADH_2_ /NADH ratio in the valproate-treated livers. However, an increase in FADH_2_ /NADH ratio is known to elevate reactive oxygen species (ROS) production, resulting in the impairment and breakdown of mitochondrial complex I and its dissociation from the complex I / complex III supercomplex (Guaras, Perales-Clemente et al. 2016, Onukwufor, Berry et al. 2019). Supporting this, valproate has been reported to increase mitochondrial ROS levels (Pirozzi, Lama et al. 2020). In our data, we noted a profound decrease in the amounts of proteins forming or associated with complex I, such as NADH:ubiquinone oxidoreductase subunits (**A**, purple), indicating a deficiency of NADH-driven electron supply to the electron transport chain. Furthermore, valproate, which is catabolized by mitochondria via β-oxidation, has the potential to disrupt mitochondrial FAO by impeding several key FAO enzymes (Silva, Aires et al. 2008). If FAO inhibition had indeed occurred in the valproate group, the observed up-regulation of FAO-associated mitochondrial proteins (**B**) could potentially be a compensatory feedback mechanism aiming to support the pathway efficiency. Taken together, the observed changes in the proteome of valproate-treated livers suggest major deficiency in mitochondrial respiration with both pyruvate- and fatty acid-oxidation being compromised. This has resulted in mitochondrial restructuring aiming to compensate for the losses in electron donors and provide an adequate energy supply.

It is important to note that, in addition to mitochondrial FAO, β-oxidation in peroxisomes can be an additional source of energy supply, in the form of acylcarnitine, composed of partially oxidized, chain-shortened FA, which are more ‘digestible’ for mitochondria (Chornyi, IJlst et al. 2020, Houten, Wanders et al. 2020). There is a possibility, not confirmed experimentally, that peroxisomes can feed mitochondria with NADH, which has been shown to shuttle from peroxisomes to mitochondria in yeasts (Fransen, Lismont et al. 2017). Strikingly, peroxisomal β-oxidation, unlike mitochondrial FAO, is not inhibited by valproate. The size of peroxisomal pool and activity of the key peroxisomal enzymes are known to increase upon continuous valproate administration (Ponchaut, Draye et al. 1991), and in agreement, we observed increased levels of Acaa1, Acox1, Hsd17b4 and other peroxisomal proteins (**B**, dark grey). The underlying mechanisms are not clear; however, mitochondrial deficiency in FAO and hyperproduction of ROS (levels of mitochondrial ROS scavengers Sod2, Txn2, and Txnrd2 are elevated, not shown) can contribute to the activation of peroxisomal FA oxidation due to the efficient interconnection between mitochondrial and peroxisomal machineries (Fransen, Lismont et al. 2017). Along with FA processing, peroxisomes play a crucial role in both the synthesis and conjugation of bile acids (Ferdinandusse, Denis et al. 2009), and the observed alterations in peroxisomal function, including cholesterol metabolism, support the activation of the hepatic BA synthesis in valproate-treated animals (Fig. 1B and Fig. 4C).


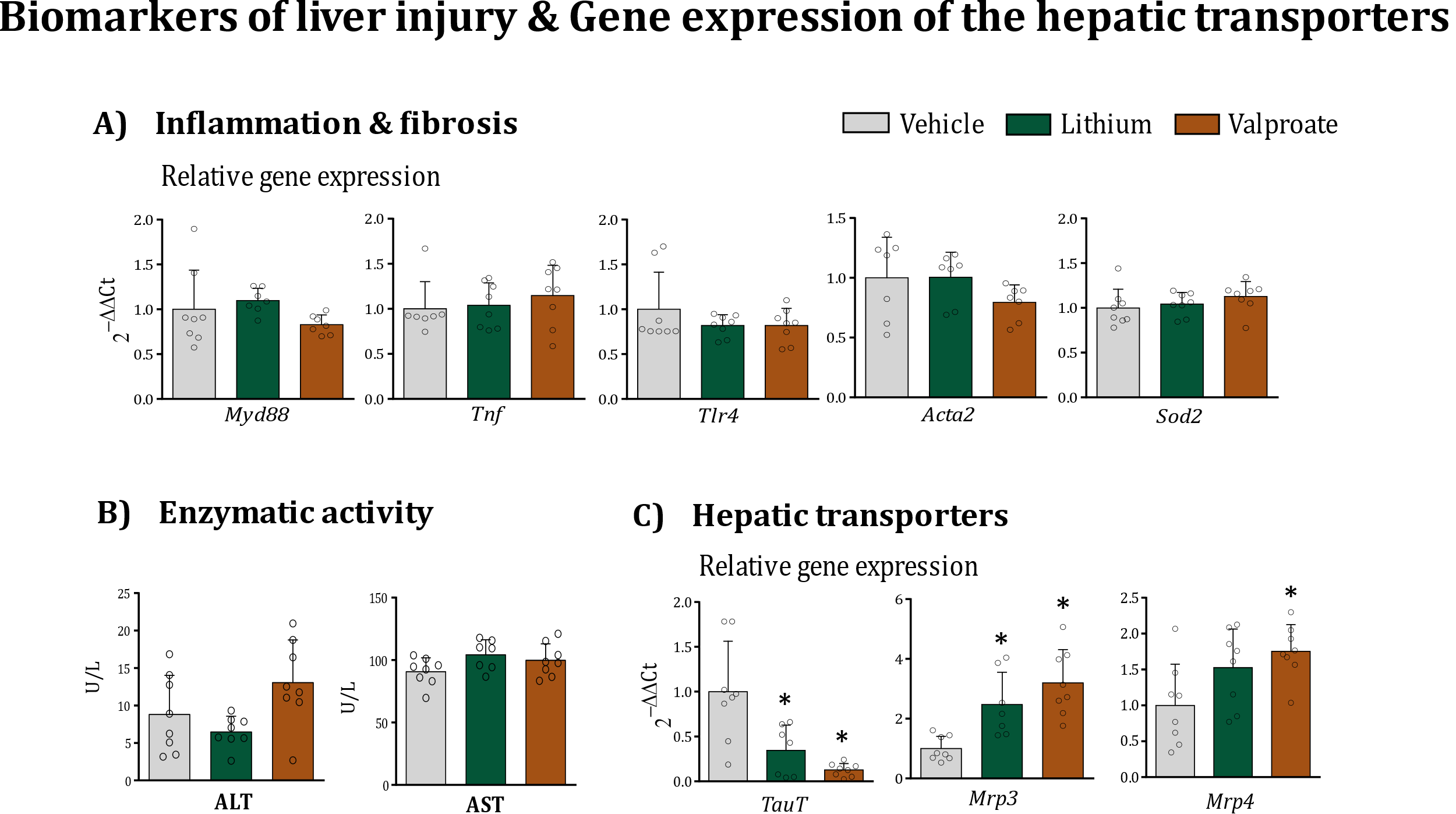


**Figure S6. (A-B) Liver tissues in lithium- or valproate-treated animals did not display evidence of hepatotoxicity**. **(A)** Neither drug affected the gene expression of inflammatory and fibrosis markers. **(B)** Alanine aminotransferase (ALT) and aspartate transaminase (AST) enzymatic activities in plasma were not impacted by either treatment. **(C) Lithium and valproate affected the gene expression of hepatic transporters.** Both drugs down-regulated the secondary transporter of taurine TauT and up-regulated the multidrug resistance-associated protein (Mrp) 3/4 transporters that mediate the alternative basolateral efflux of bile to the sinusoidal blood.

Statistical details: Data are presented as mean ± SD. *p<0.05 (n=7-8/group). For *Tlr4* and *Acta2*: Kruskal-Wallis (KW) test followed by Mann-Whitney (MW). *Tlr4*: KW χ^2^_(2)_=0.095, *p*=0.954. *Acta2*: KW χ^2^_(2)_=2.7, *p*=0.259. For *Mrp3, Mrp4, Myd88, TauT, Tnf-α, Sod2*, ALT, AST: One-way ANOVA followed by Dunnett’s posthoc. *Mrp3*: F_(2,22)_=11.83, *p*=0.000; vs Lit *p*=0.011, vs Val *p*=0.000. *Mrp4*: F_(2,23)_=4.74, *p*=0.020; vs Lit *p*=0.086, vs Val *p*=0.013. *Myd88*: F_(2,21)_=1.59, *p*=0.229. *TauT*: F_(2,22)_=12.01, *p*=0.000; vs Lit *p*=0.005, vs Val *p*=0.000. *Tnf-α*: F_(2,22)_=0.504, *p*=0.611. *Sod2*: F_(2,23)_=1.15, *p*=0.336. ALT: F_(2,22)_=2.43, *p*=0.036; vs Lit *p*=0.713, vs Val *p*=0.162. AST: F_(2,22)_=3.17, *p*=0.011; vs Lit *p*=0.127, vs Val *p*=0.432.


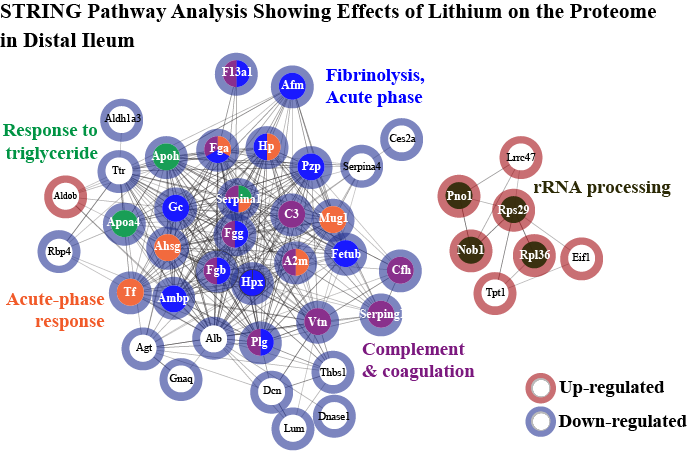


**Figure S7**. **STRING pathway analysis showing effects of lithium on the proteome in distal ileum.** Only significantly affected (FDR adjusted p<0.05) proteins with an effect size >│0.5│on the log2-fold scale were included in the analysis. See **Table S7** for statistical details. Chronic treatment with lithium significantly changed the expression of 301 proteins in distal ileum tissue, with 143 proteins being up-regulated and 158 being down-regulated by lithium. We further selected the proteins with the strongest effect size (EE >0.5 or <-0.5 on the log2-fold scale) which narrowed the list to 39 up-regulated and 65 down-regulated proteins. For functional enrichment analysis, these proteins were uploaded to STRING (https://string-db.org/). The strongest significantly implicated (FDR adjusted p<0.05) protein pathways were colour-coded in the generated protein interaction network. Red and blue halos indicate significantly increased and decreased protein content, respectively. The analysis revealed a large cluster of 36 mostly downregulated proteins associated with coagulation, complement, and acute phase responses. These proteins were most likely coming from blood vessels.

| **Colour** | **Pathway source** | **Pathway ID** | **Pathway Name** | **Count in Network** | **Strength** | **P value after FDR** |
| --- | --- | --- | --- | --- | --- | --- |
| **BLUE** | STRING | CL:17864 | *Mixed, incl. Fibrinolysis, and Acute phase* | 13 of 23 | 2.13 | 7.09E-20 |
| **PURPLE** | KEGG | rno04610 | *Complement and coagulation cascades* | 11 of 78 | 1.53 | 3.30E-11 |
| **ORANGE** | Gene Ontology | GO:0006953 | *Acute-phase response* | 7 of 43 | 1.59 | 2.73E-06 |
| **GREEN** | Gene Ontology | GO:0034014 | *Response to triglyceride* | 3 of 6 | 2.08 | 0.0016 |
| **DARK GREY** | Reactome | RNO-6791226 | *Major pathway of rRNA processing in the nucleolus and cytosol* | 4 of 182 | 1.84 | 0.00027 |


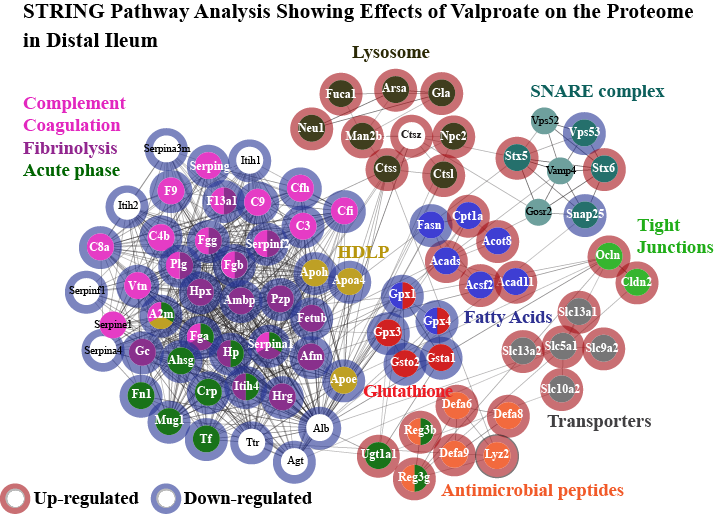


**Figure S8**. **STRING pathway analysis showing effects of valproate on the proteome in distal ileum.** Only significantly affected (FDR adjusted p<0.05) proteins with an effect size >│0.5│on the log2-fold scale were included in the analysis. See **Table S7** for statistical details. Chronic treatment with valproate significantly changed the expression of 844 proteins in distal ileum tissues, with 470 proteins being up-regulated and 374 being down-regulated by valproate. After filtering for the effect size, we reduced the list to 132 up-regulated and 121 down-regulated proteins. For functional enrichment analysis, these proteins were uploaded to STRING (https://string-db.org/). The strongest significantly implicated (FDR adjusted p<0.05) protein pathways were colour-coded in the generated protein interaction network. Red and blue halos indicate significantly increased and decreased protein content, respectively. Similar to lithium, valproate induced a down-regulation of proteins involved in blood coagulation, complement, fibrinolysis, and acute phase responses (the large cluster on the left-hand side, PINK, PURPLE, DARK GREEN, and YELLOW). Other potentially interesting pathways affected by chronic valproate treatment included: *Glutathione metabolism* (RED); *Antimicrobial peptides* (ORANGE); *Glycosphingolipid metabolism, and Lysosome* (DARG GREY); *Fatty acid metabolism* (BLUE); *SNARE complex, and Retrograde transport at the Trans-Golgi-Network* (AQUAMARINE). We’ve also noticed that valproate upregulated a few tight junction proteins (LIGHT GREEN) and apical membrane transporters, including bile acid transporter ASBT (Slc10a2), in distal ileum tissues (LIGHT GREY).

| **Colour** | **Pathway source** | **Pathway ID** | **Pathway Name** | **Count in Network** | **Strength** | **P value after FDR** |
| --- | --- | --- | --- | --- | --- | --- |
| **PINK** | KEGG | rno04610 | *Complement and coagulation cascades* | 18 of 78 | 1.32 | 5.75E-15 |
| **PURPLE** | STRING | CL:17864 | *Mixed, incl. Fibrinolysis, and Acute phase* | 16 of 23 | 1.80 | 4.77E-19 |
| **DARK GREEN** | Gene Ontology | GO:0006953 | *Acute-phase response* | 13 of 43 | 1.44 | 7.27E-11 |
| **YELLOW** | STRING | CL:18106 | *High-density lipoprotein particle, and Apolipoprotein binding* | 4 of 29 | 1.10 | 0.0497 |
| **RED** | KEGG | rno00480 | *Glutathione metabolism* | 5 of 62 | 0.86 | 0.0257 |
| **ORANGE** | Reactome | RNO-6803157 | *Antimicrobial peptides* | 8 of 67 | 1.03 | 0.00014 |
| **DARK GREY** | STRING | CL:29118 | *Glycosphingolipid metabolism, and Lysosome* | 8 of 67 | 1.03 | 0.00062 |
| **BLUE** | Reactome | RNO-8978868 | *Fatty acid metabolism* | 8 of 162 | 0.65 | 0.0187 |
| **AQUAMARINE** | STRING | CL:24066 | *SNARE complex, and Retrograde transport at the Trans-Golgi-Network* | 8 of 88 | 0.91 | 0.0029 |


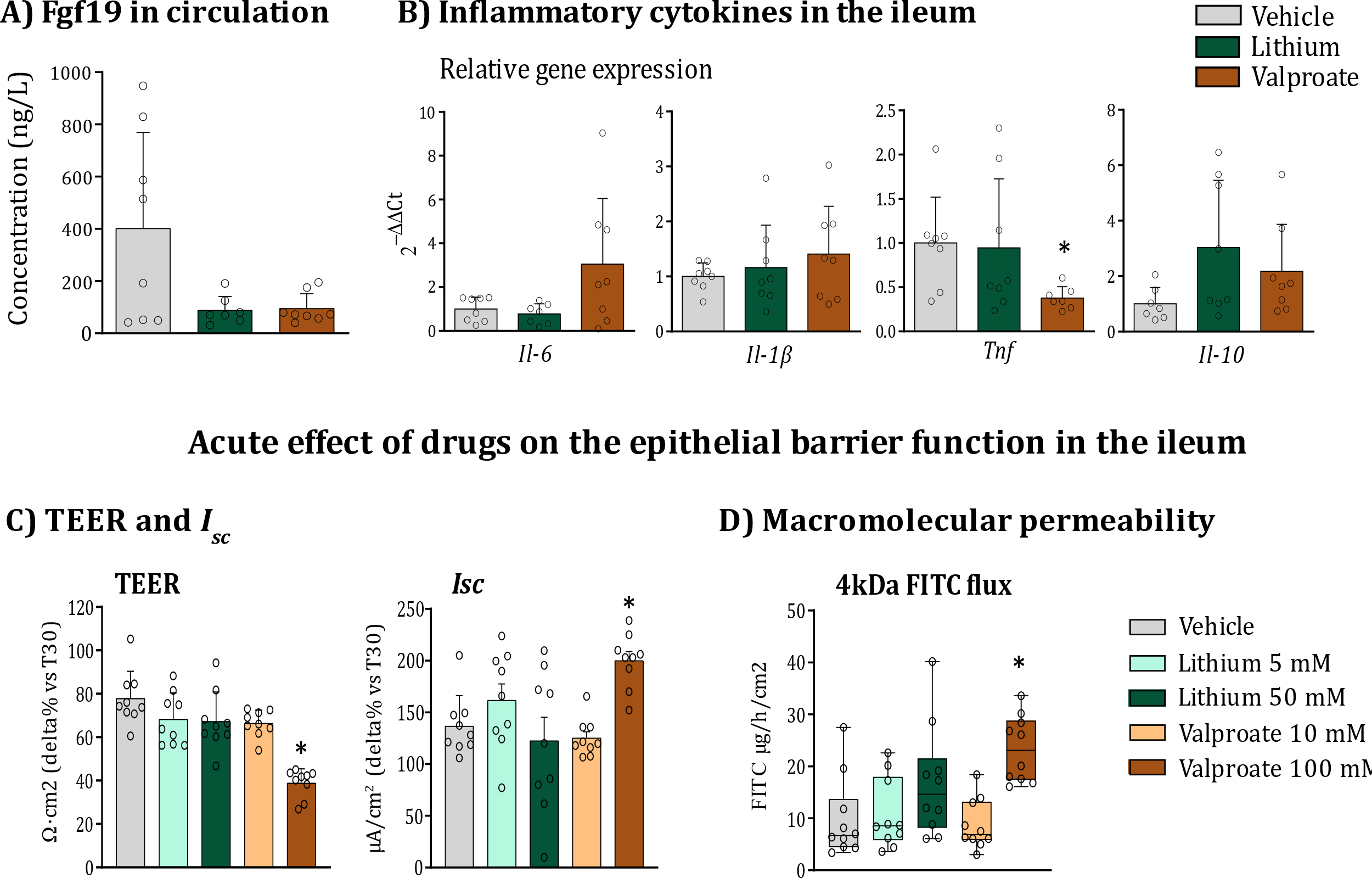


**Figure S9. (A) Lithium and valproate decreased the circulating levels of Fgf19 in chronically treated animals.** Fgf19 levels were reduced compared to the vehicle group, although this difference did not reach statistical significance due to the high variance in the control group. **(B)** **Lithium and valproate had marginal effects on the small intestine inflammatory markers in chronically treated animals**. The expression of *Il-1β*, *Il-6*, and *Il-10* genes were similar across groups. Valproate decreased the expression of *Tnfa*. **(C-D)** **Dose-dependent effects of lithium and valproate on epithelial permeability in distal ileum upon acute treatment in Ussing chambers.** Acute administration of lithium in 5 mM and 50 mM concentrations had no effect on TEER and *Isc* (C), as well as on macromolecular permeability of the intestine to 4kDa FITC marker (D). Acute administration of valproate in 100 mM, but not in 10 mM concentration decreased TEER, increased *I_sc_* (C), as well as increased epithelial permeability to 4kDa FITC.

***Statistical details***: (A-B) Data are presented as mean ± SD. ^*^*p*<0.05 (n=7-8/group). For *Il1b*, One-way ANOVA followed by Dunnett’s posthoc. *Il1b*: F_(2;23)_=0.718, p=0.499. For *Il6,* *Tnf,* and *Il10*, Kruskal-Wallis (KW) test followed by Mann-Whitney (MW). *Il6*: KW χ^2^_(2)_=3.963, *p*=0.138; *Tnf*: KW χ^2^_(2)_=6.504, *p*=0.039, MW vs Lit p=1.00, vs Val p=0.41; *Il10*: KW χ^2^_(2)_=4.400, *p*=0.111. (C) Data are presented as mean ± SD. ^*^*p*<0.05 (n=9/group). One-way ANOVA followed by Dunnett’s posthoc. TEER: F_(4;44)_=16.95, p=0.001; vs Lit5 *p*=0.19, vs Lit50 *p*=0.13, vs Val10 *p*=0.08, vs Val100 *p*=0.000. *I_sc_*: F_(4;44)_=5.38, p=0.001; vs Lit5 *p*=0.66, vs Lit50 *p*=0.98, vs Val10 *p*=0.85, vs Val100 *p*=0.002. (D) Data are presented as median, IQR + min-to-max values. ^*^*p*<0.05 (n=10/group). Kruskal-Wallis (KW) test followed by Mann-Whitney (MW). KW χ^2^_(4)_=17.28, *p*=0.002, MW vs Lit5 *p*=0.45; vs Lit50 *p*=0.076; vs Val10 *p*=0.94; vs Val100 *p*=0.003.


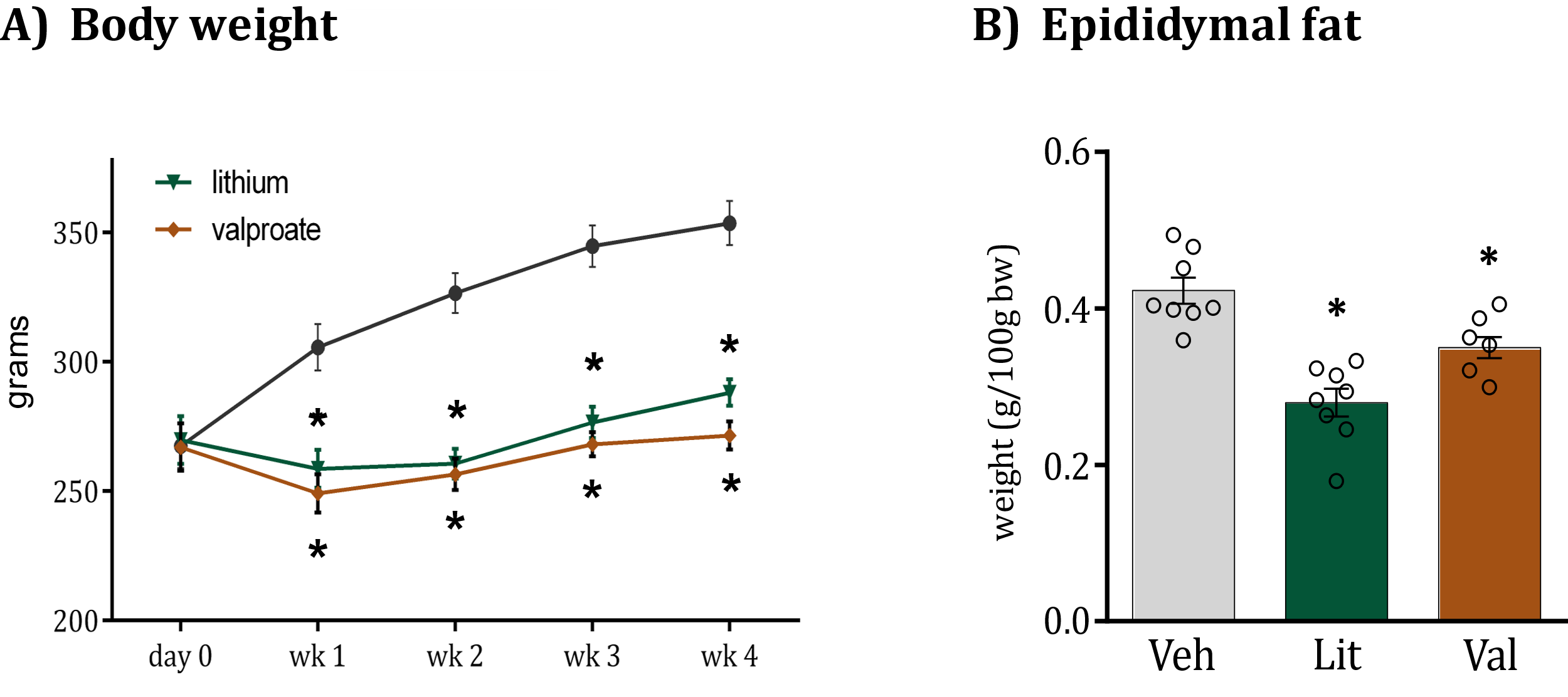


**Figure S10. Changes in body weight and fat deposition in lithium- and valproate-treated animals. (A)** Administration of lithium and valproate significantly decreased body weight. A mixed between-within subjects ANOVA revealed a significant effect for Time [F_(4;196)_=250.06, *p*<0.001], Treatment [F_(6;49)_=14.15, *p*<0.001] and a Time × Treatment interaction [F_(24;196)_=12.75, *p*<0.001]. P<0.001 for both drugs on weeks 1-4 (all p<0.001). **(B)** Both drugs decreased epididymal white adipose tissue deposition (g/100g of body weight, one-way ANOVA F_(6;55)_=13.237, *p*=0.000; post-hoc *p*=0.000 for lithium and *p*=0.020 for valproate). Data are expressed as mean ± SEM. **p*<0.05 (n=8/group).


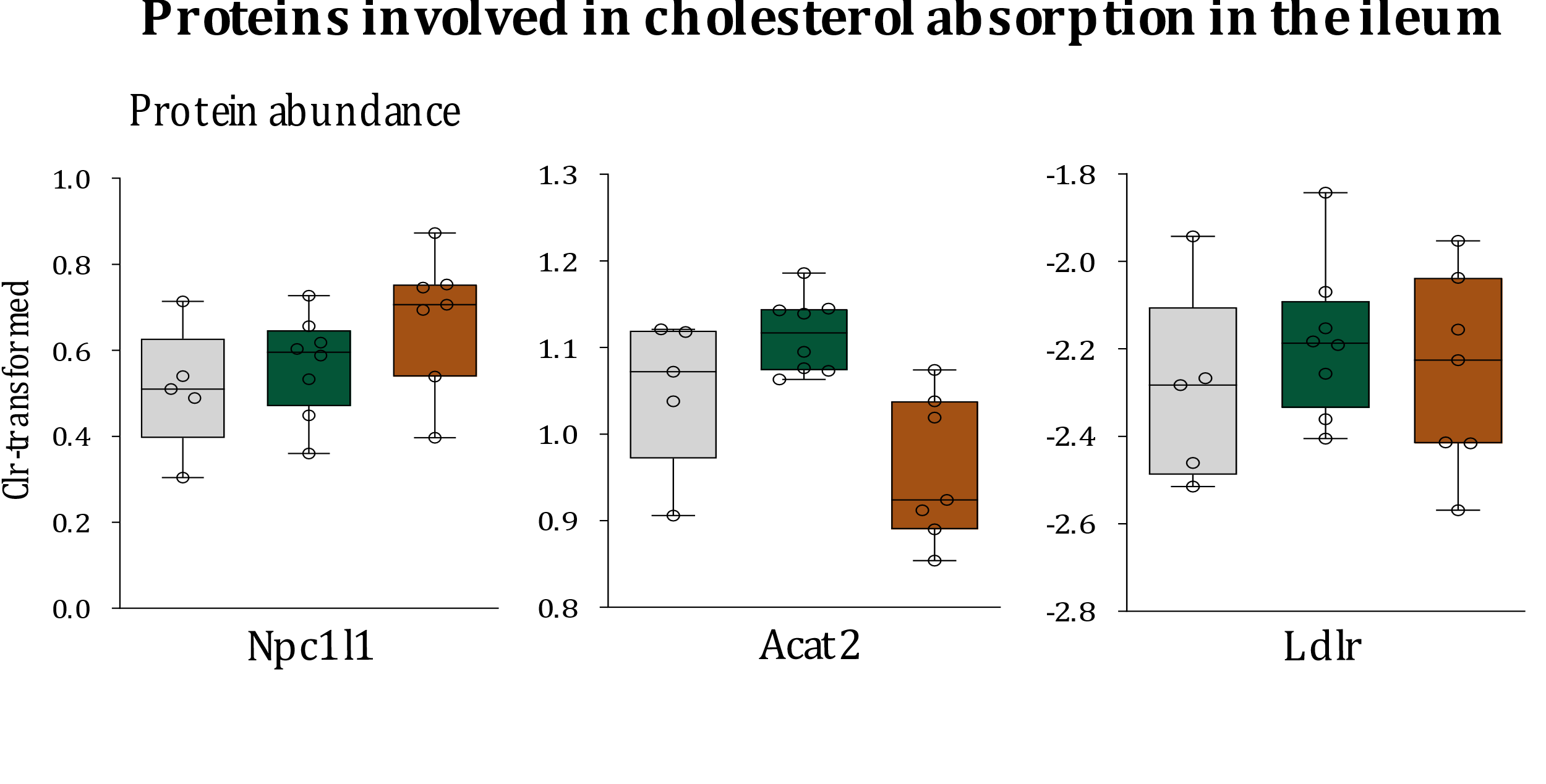


**Figure S11. Lithium and valproate had no effect on the expression of proteins involved in the intestinal absorption of cholesterol. (A)** NPC1-like intracellular cholesterol transporter 1 (Npc1l1) is critical for the uptake of cholesterol from the gut lumen. One-way ANOVA F_(2;20)_=2.146, *p*=0.148. **(B)** Acetyl-CoA acetyltransferase, cytosolic (Acat2) esterifies cholesterol in the endoplasmic reticulum of enterocytes, facilitating the absorption. One-way ANOVA F_(2;20)_=8.938, *p*=0.002; post-hoc p=0.221 for lithium and p=0.072 for valproate. **(C)** Low-density lipoprotein receptor (Ldlr) binds LDL, the major cholesterol-carrying lipoprotein of plasma, and transports it into cells by endocytosis. One-way ANOVA F_(2;20)_=0.491, *p*=0.620. Data are presented as median, IQR + min-to-max values.

**References**

1. Chornyi, S., L. IJlst, C. van Roermund, R. Wanders and H. Waterham (2020). Peroxisomal metabolite and cofactor transport in humans. Front Cell Dev Biol 8: 613892.
2. Ferdinandusse, S., S. Denis, P. L. Faust and R. J. Wanders (2009). "Bile acids: the role of peroxisomes." J Lipid Res **50**(11): 2139-2147.
3. Fransen, M., C. Lismont and P. Walton (2017). "The peroxisome-mitochondria connection: how and why?" International journal of molecular sciences **18**(6): 1126.
4. Guaras, A., E. Perales-Clemente, E. Calvo, R. Acín-Pérez, M. Loureiro-Lopez, C. Pujol, I. Martínez-Carrascoso, E. Nuñez, F. García-Marqués and M. A. Rodríguez-Hernández (2016). "The CoQH2/CoQ ratio serves as a sensor of respiratory chain efficiency." Cell reports **15**(1): 197-209.
5. Houten, S. M., R. J. Wanders and P. Ranea-Robles (2020). "Metabolic interactions between peroxisomes and mitochondria with a special focus on acylcarnitine metabolism." Biochimica et Biophysica Acta (BBA)-Molecular Basis of Disease **1866**(5): 165720.
6. Kudin, A. P., H. Mawasi, A. Eisenkraft, C. E. Elger, M. Bialer and W. S. Kunz (2017). "Mitochondrial liver toxicity of valproic acid and its acid derivatives is related to inhibition of α-lipoamide dehydrogenase." International Journal of Molecular Sciences **18**(9): 1912.
7. Luis, P. B., J. P. Ruiter, C. C. Aires, G. Soveral, I. T. de Almeida, M. Duran, R. J. Wanders and M. F. Silva (2007). "Valproic acid metabolites inhibit dihydrolipoyl dehydrogenase activity leading to impaired 2-oxoglutarate-driven oxidative phosphorylation." Biochim Biophys Acta **1767**(9): 1126-1133.
8. Onukwufor, J. O., B. J. Berry and A. P. Wojtovich (2019). "Physiologic implications of reactive oxygen species production by mitochondrial complex I reverse electron transport." Antioxidants **8**(8): 285.
9. Pettersen, I. K. N., D. Tusubira, H. Ashrafi, S. E. Dyrstad, L. Hansen, X.-Z. Liu, L. I. H. Nilsson, N. G. Løvsletten, K. Berge and H. Wergedahl (2019). "Upregulated PDK4 expression is a sensitive marker of increased fatty acid oxidation." Mitochondrion **49**: 97-110.
10. Pirozzi, C., A. Lama, C. Annunziata, G. Cavaliere, C. De Caro, R. Citraro, E. Russo, M. Tallarico, M. Iannone and M. C. Ferrante (2020). "Butyrate prevents valproate‐induced liver injury: in vitro and in vivo evidence." The FASEB Journal **34**(1): 676-690.
11. Ponchaut, S., J. P. Draye, K. Veitch and F. Van Hoof (1991). "Influence of chronic administration of valproate on ultrastructure and enzyme content of peroxisomes in rat liver and kidney: Oxidation of valproate by liver peroxisomes." Biochemical pharmacology **41**(10): 1419-1428.
12. Silva, M., C. Aires, P. Luis, J. Ruiter, L. IJlst, M. Duran, R. Wanders and I. Tavares de Almeida (2008). "Valproic acid metabolism and its effects on mitochondrial fatty acid oxidation: a review." Journal of inherited metabolic disease **31**: 205-216.
13. Silva, M. F., J. P. Ruiter, L. Illst, C. Jakobs, M. Duran, I. T. de Almeida and R. J. Wanders (1997). "Valproate inhibits the mitochondrial pyruvate-driven oxidative phosphorylation in vitro." J Inherit Metab Dis **20**(3): 397-400.
14. Woolbright, B. L. and R. A. Harris (2021). "PDK2: An Underappreciated Regulator of Liver Metabolism." Livers **1**(2): 82-97.
