# Supplementary material for: The mood stabilizers lithium and valproate disrupt hepatic and intestinal farnesoid X receptor signalling and increase bile synthesis in the rat
Source: Exp Physiol. 2025 Mar 28;110(9):1233–53. doi: 10.1113/EP092451 (PMC12400835; doi:10.1113/EP092451)
Supplement: Supplementary file 3 — Supplementary Tables S1–S4. [file EPH-110-1233-s004.docx]

**SUPPLEMENTARY TABLES S1-S4**

**Table S1. Bile acids levels in the plasma of rats treated with lithium and valproate (ng/mL)**

| **Bile acid (full name)** | **Abbreviation** | **Vehicle**  **Median (IQR)** | **Lithium**  **Median (IQR) [(i/m)Q]** | **Valproate**  **Median (IQR) [(i/m)Q]** |
| --- | --- | --- | --- | --- |
| **Unconjugated bile acids** | | | | |
| α/ω-Muricholic acid | α/ω-MCA | 513.6 (548.1) | 9711 (16567) [0.0138] * | 6041 (7952) [0.0086] * |
| β-Muricholic acid | βMCA | 435.8 (557.3) | 1987 (2684) [0.0396] * | 2995 (3603) [0.0103] * |
| Chenodeoxycholic acid | CDCA | 414.4 (544.4) | 7213 (7586) [0.0017] * | 1993 (3026.6) [0.0345] * |
| Cholic acid | CA | 2032 (2101) | 15342 (11906) [0.0034] * | 20198 (18004) [0.0017] * |
| Deoxycholic acid | DCA | 381.2 (402.1) | 1857 (1682) [0.0086] * | 1012 (1440) [0.0327] * |
| Hyocholic acid | HCA | 215.6 (244.8) | 1722 (1096) [0.0121] * | 1404 (1208) [0.0069] * |
| Hyodeoxycholic acid | HDCA | 63.24 (119.03) | 671.7 (2174.6) [0.0103] * | 186.4 (343.48) [0.0431] * |
| Lithocholic acid | LCA | 13.13 (3.684) | 108 (349.14) [0.0052] * | 35.08 (43.96) [0.0034] * |
| Ursodeoxycholic acid | UDCA | 34.51 (19.01) | 486.8 (935.3) [0.0069] * | 378.7 (310.8) [0.0052] * |
| **Tauro-conjugated bile acids** | | | | |
| Taurochenodeoxycholic acid | TCDCA | 65.99 (33.46) | 87.11 (61.78) | 41.12 (22.48) [0.0396] * |
| Taurocholic acid | TCA | 233.4 (153.2) | 92.6 (156.05) | 218.9 (140.1) |
| Taurodeoxycholic acid | TDCA | 84.07 (60.35) | 24.5 (21.51) [0.0448] * | 19.95 (20.88) [0.0362] * |
| Taurohyocholic acid | THCA | 2.257 (2.755) | 1.528 (1.381) | 2.013 (3.811) |
| Taurohyodeoxycholic acid | THDCA | 8.422 (4.441) | 8.977 (3.583) | 6.213 (4.127) |
| Taurolithocholic acid | TLCA | 1.678 (0.644) | 5.707 (2.002) [0.0172] * | 1.573 (1.63) |
| Tauromuricholic acid | TMCA | 367.2 (231.9) | 382.4 (322) | 349.4 (149.8) |
| Tauroursodeoxycholic acid | TUDCA | 8.32 (4.067) | 7.79 (11.386) | 4.97 (2.663) |
| **Glyco-conjugated bile acids** | | | | |
| Glycochenodeoxycholic acid | GCDCA | 5.104 (6.969) | 275.9 (443.94) [0.0189] * | 52.01 (81.08) [0.0396] * |
| Glycocholic acid | GCA | 112.2 (116.81) | 819.5 (1092.7) [0.0207] * | 748.9 (339.7) [0.0155] * |
| Glycodeoxycholic acid | GDCA | 12.33 (18.624) | 246 (116.3) [0.0224] * | 92.31 (115.01) [0.0172] * |
| Glycohyocholic acid | GHCA | 5.432 (11.405) | 29.33 (37.12) [0.0431] * | 8.545 (12.792) |
| Glycolithocholic acid | GLCA | 0.7683 (0.674) | 6.587 (7.767) [0.0345] * | 1.317 (0.988) |
| Glycoursodeoxycholic acid | GUDCA | 2.53 (6.47) | 23.93 (22.62) [0.0362] * | 9.652 (10.677) [0.0414] * |
|  | | | | |
| **Taurine (ng/mL)** |  | 1146 (377.6) | 856.7 (156.5) [*p*=0.010] # | 915.8 (211.7) [*p*=0.042] # |

*Data that did not satisfy the criteria of homogeneity of variances and/or normal distribution:* ^*^*p*<0.05, Kruskal-Wallis nonparametric test followed by Mann-Whitney U test. Reported is the Benjamini-Hochberg critical value [(i/m)Q] with a Q (false-discovery rate) of 0.05. *Data that satisfied the criteria of homogeneity of variances and normal distribution:* ^#^*p*<0.05 one-way ANOVA. Reported is the *p*-value of Dunnett’s post-hoc test *vs* vehicle. N=7-8/group. Red indicates an increase, and blue indicates a decrease in bile acid levels in treatment groups compared to the vehicle group.

**Table S2. Bile acids levels in the liver of rats treated with lithium and valproate (ng/mg)**

| **Bile acid (full name)** | **Abbreviation** | **Vehicle**  **Median (IQR)** | **Lithium**  **Median (IQR) [(i/m)Q]** | **Valproate**  **Median (IQR) [(i/m)Q]** |
| --- | --- | --- | --- | --- |
| **Unconjugated bile acids** | | | | |
| α/ω-Muricholic acid | α/ω-MCA | 0.287 (0.3761) | 2.651 (16.155) [0.0083] * | 5.090 (2.871) [0.0117] * |
| β-Muricholic acid | βMCA | 0.3029 (1.0848) | 1.552 (7.5656) [0.0450] * | 5.471 (5.31) [0.0133] * |
| Chenodeoxycholic acid | CDCA | 0.02844 (0.0364) | 0.2859 (0.674) [0.0033] * | 0.1207 (0.2396) [0.0033] |
| Cholic acid | CA | 0.649 (0.7545) | 1.906 (3.867) [0.0433] * | 9.087 (9.209) [0.0050] * |
| Deoxycholic acid | DCA | 0.06368 (0.0728) | 0.1332 (0.1108) [*p*=0.026] # | 0.1599 (0.1121) [*p*=0.022] # |
| Hyocholic acid | HCA | 0.2151 (0.2957) | 1.060 (1.2973) [0.0350] * | 3.405 (4.893) [0.0100] * |
| Hyodeoxycholic acid | HDCA | 0.0091 (0.0089) | 0.087 (0.3059) [0.0050] * | 0.1012 (0.0464) [0.0067] * |
| Lithocholic acid | LCA | 0.00546 (0.00284) | 0.01052 (0.00769) [*p*=0.010] # | 0.0086 (0.00618) |
| Ursodeoxycholic acid | UDCA | 0.02014 (0.0158) | 0.1070 (0.4678) [0.0383] * | 0.04171 (0.0342) [0.0467] * |
| **Tauro-conjugated bile acids** | | | | |
| Taurochenodeoxycholic acid | TCDCA | 9.301 (4.719) | 16.08 (6.45) [*p*=0.001] # | 4.019 (4.184) [*p*=0.014] # |
| Taurocholic acid | TCA | 68.31 (4.07) | 41.50 (17.7) [*p*=0.000] # | 32.95 (5.18) [*p*=0.000] # |
| Taurodeoxycholic acid | TDCA | 11.17 (5.112) | 4.767 (5.161) [*p*=0.000] # | 2.761 (2.076) [*p*=0.000] # |
| Taurohyocholic acid | THCA | 0.2239 (0.0808) | 0.2686 (0.1989) | 0.07879 (0.0242) [0.0283] * |
| Taurohyodeoxycholic acid | THDCA | 0.5304 (0.2369) | 0.8630 (0.6109) [0.0467] * | 0.2201 (0.122) [0.0183] * |
| Taurolithocholic acid | TLCA | 0.06486 (0.0479) | 0.3092 (0.1653) [0.0117] * | 0.03216 (0.0503) [0.0500] * |
| Tauromuricholic acid | TMCA | 97.42 (49.19) | 76.75 (17.78) [*p*=0.039] # | 37.21 (23.48) [*p*=0.000] # |
| Tauroursodeoxycholic acid | TUDCA | 2.12 (0.308) | 2.884 (1.179) [*p*=0.028] # | 0.5649 (0.4501) [*p*=0.000] # |
| **Glyco-conjugated bile acids** | | | | |
| Glycochenodeoxycholic acid | GCDCA | 0.1075 (0.0184) | 14.70 (10.432) [0.0150] * | 1.795 (2.219) [0.0300] * |
| Glycocholic acid | GCA | 3.046 (3.084) | 64.05 (23.26) [0.0167] * | 26.15 (10.22) [0.0317] * |
| Glycodeoxycholic acid | GDCA | 0.2062 (0.1313) | 8.677 (4.681) [0.0200] * | 2.764 (3.624) [0.0350] * |
| Glycohyocholic acid | GHCA | 0.0564 (0.0168) | 4.132 (3.11) [0.0233] * | 0.5586 (0.6213) [0.0383] * |
| Glycolithocholic acid | GLCA | 0.00161 (0.00145) | 0.1066 (0.1591) [0.0133] * | 0.01096 (0.0112) [0.0200] * |
| Glycoursodeoxycholic acid | GUDCA | 0.02697 (0.0056) | 1.781 (2.139) [0.0183] * | 0.2489 (0.252) [0.0333] * |
|  | | | | |
| **Taurine (ng/mg)** |  | 85.94 (61.4) | 13.09 (5.02) [0.0017] * | 16.11 (3.5) [0.0017] * |

*Data that did not satisfy the criteria of homogeneity of variances and/or normal distribution:* ^*^*p*<0.05, Kruskal-Wallis nonparametric test followed by Mann-Whitney U test. Reported is the Benjamini-Hochberg critical value [(i/m)Q] with a Q (false-discovery rate) of 0.05. *Data that satisfied the criteria of homogeneity of variances and normal distribution:* ^#^ *p*<0.05 one-way ANOVA. Reported is the *p*-value of Dunnett’s post-hoc *vs* vehicle. N=7-8/group. Red indicates an increase, and blue indicates a decrease in bile acid levels in treatment groups compared to the vehicle group.

**Table S3. Bile acids levels in the faeces of rats treated with lithium and valproate (ng/mg)**

| **Bile acid (full name)** | **Abbreviation** | **Vehicle**  **Median (IQR)** | **Lithium**  **Median (IQR) [(i/m)Q]** | **Valproate**  **Median (IQR) [(i/m)Q]** |
| --- | --- | --- | --- | --- |
| **Unconjugated bile acids** | | | | |
| α/ω-Muricholic acid | α/ω-MCA | 433.2 (215.9) | 637.3 (330.7) [*p*=0.041] # | 664.8 (520.9) [*p*=0.035] # |
| β-Muricholic acid | βMCA | 113 (65.21) | 127.7 (61.6) | 241.5 (293.3) [0.0458] * |
| Chenodeoxycholic acid | CDCA | 2.7 (2.767) | 9.543 (10.384) [0.0251] * | 7.089 (16.311) [0.0375] * |
| Cholic acid | CA | 2.562 (1.775) | 6.28 (2.788) [0.0319] * | 8.093 (6.326) [0.0250] * |
| Deoxycholic acid | DCA | 220.7 (107.5) | 436.2 (167.2) [0.0028] * | 393.2 (411.2) [0.0417] * |
| Hyocholic acid | HCA | 3.797 (6.005) | 8.722 (5.454) | 3.214 (5.675) |
| Hyodeoxycholic acid | HDCA | 13.53 (21.901) | 52.62 (27.09) [0.0347] * | 37.79 (9.94) [0.0306] * |
| Lithocholic acid | LCA | 13.66 (4.2) | 65.87 (39.97) [0.0014] * | 18.61 (10.34) [0.0444] * |
| Ursodeoxycholic acid | UDCA | 2.407 (1.126) | 12.4 (11.112) [0.0333] * | 1.762 (8.361) |
| **Tauro-conjugated bile acids** | | | | |
| Taurochenodeoxycholic acid | TCDCA | 0.7492 (0.3443) | 0.5961 (0.4235) | 0.1601 (0.2484) [0.0208] * |
| Taurocholic acid | TCA | 4.29 (1.667) | 0.6898 (0.0873) [0.0056] * | 0.7219 (0.4819) [0.0028] * |
| Taurodeoxycholic acid | TDCA | 2.102 (0.767) | 0.4165 (0.3691) [0.0069] * | 0.1697 (0.1481) [0.0056] * |
| Taurohyocholic acid | THCA | 0.1386 (0.0728) | 0.1012 (0.0285) | 0.1479 (0.0847) |
| Taurohyodeoxycholic acid | THDCA | 0.1761 (0.069) | 0.1148 (0.0674) [0.0403] * | 0.0824 (0.0157) [0.0069] * |
| Taurolithocholic acid | TLCA | 0.1822 (0.0803) | 0.1211 (0.0837) | 0.0366 (0.0155) [0.0042] * |
| Tauromuricholic acid | TMCA | 12.07 (6.861) | 2.392 (1.12) [0.0083] * | 1.943 (1.917) [0.0083] * |
| Tauroursodeoxycholic acid | TUDCA | 0.4522 (0.1541) | 0.2738 (0.1542) | 0.1407 (0.0614) [0.0181] * |
| **Glyco-conjugated bile acids** | | | | |
| Glycochenodeoxycholic acid | GCDCA | 0.0477 (0.0153) | 0.7781 (0.1506) [0.0278] * | 0.2149 (0.2142) [0.0208] * |
| Glycocholic acid | GCA | 0.3905 (0.5762) | 2.606 (0.819) [0.0097] * | 2.287 (1.276) [0.0222] * |
| Glycodeoxycholic acid | GDCA | 0.0664 (0.035) | 0.3664 (0.1206) [0.0292] * | 0.2606 (0.426) [0.0278] * |
| Glycohyocholic acid | GHCA | 0.0846 (0.024) | 0.1409 (0.0637) [0.0417] * | 0.1788 (0.061) [0.0292] * |
| Glycolithocholic acid | GLCA | 0.01995 (0.0058) | 0.0910 (0.0438) [0.0111] * | 0.0334 (0.0342) [0.0389] * |
| Glycoursodeoxycholic acid | GUDCA | 0.03471 (0.0332) | 0.1239 (0.0901) [0.0125] * | 0.06932 (0.0191) [0.0264] * |
|  | | | | |
| **Taurine (ng/mg)** |  | 54.31 (60.8) | 4.764 (6.567) [0.0444] * | 4.557 (2.114) [0.0472] * |

*Data that did not satisfy the criteria of homogeneity of variances and/or normal distribution:* ^*^*p*<0.05, Kruskal-Wallis nonparametric test followed by Mann-Whitney U test. Reported is the Benjamini-Hochberg critical value [(i/m)Q] with a Q (false-discovery rate) of 0.05. *Data that satisfied the criteria of homogeneity of variances and normal distribution:* ^#^*p*<0.05, one-way ANOVA. Reported is the *p-*value of Dunnett’s post-hoc *vs* vehicle. N=7-8/group. Red indicates an increase, and blue indicates a decrease in bile acid levels in treatment groups compared to the vehicle group.

**Table S4. *Ex vivo* bile acid absorption in distal ileum.** Descriptive statistics of bile acid levels detected on the serosal side of the chamber. On the luminal side, tissue was exposed to a bile mixture of known composition and treated with either linerixibat, lithium, or valproate.

| **Bile acid (full name)** | **Abbreviation** | **Vehicle**  **Median (IQR)** | **Linerixibat**  **Median (IQR)** | **Lithium 5 mM**  **Median (IQR)** | **Lithium 50 mM**  **Median (IQR)** | **Valproate 10 mM**  **Median (IQR)** | **Valproate 100 mM**  **Median (IQR)** |
| --- | --- | --- | --- | --- | --- | --- | --- |
| **Unconjugated bile acids** | | | | | | | |
| α/ω-Muricholic acid | α/ω-MCA | 14,9 (22,73) | 6,09 (9,96) | 11,51 (8,84) | 17,52 (13,75) | 8,16 (14,64) | 7,68 (8,09) |
| β-Muricholic acid | β MCA | 5,58 (5,54) | 3,03 (2,13) | 3,79 (6,28) | 5,21 (4,23) | 3,14 (8,91) | 2,95 (1,43) |
| Chenodeoxycholic acid | CDCA | 4,82 (3,41) | 3,11 (1,87) | 3,75 (2,64) | 3,83 (2,34) | 3,97 (4,64) | 2,79 (2,63) |
| Cholic acid | CA | 77,42 (148,32) | 35,36 (68,21) | 97,06 (61,39) | 132,00 (84,53) | 69,52 (132,10) | 65,32 (85,92) |
| Deoxycholic acid | DCA | 5,3 (2,13) | 5,19 (1,89) | 5,20 (1,02) | 6,21 (3,86) | 6,49 (3,55) | 5,71 (1,18) |
| Hyocholic acid | HCA | 13,46 (10,17) | 5,13 (4,18) | 10,8 (7,36) | 9,36 (8,78) | 7,04 (9,97) | 4,36 (5,36) |
| Hyodeoxycholic acid | HDCA | 2,05 (2,53) | 1,30 (1,26) | 2,1 (1,95) | 3,22 (1,73) | 2,35 (2,99) | 1,70 (1,35) |
| Lithocholic acid | LCA | 0,79 (1,01) | 0,49 (0,64) | 0,97 (0,87) | 1,00 (0,72) | 1,43 (0,74) | 1,25 (0,55) |
| Ursodeoxycholic acid | UDCA | 0,34 (0,17) | 0,30 (0,15) | 0,51 (0,5) | 0,48 (0,34) | 0,37 (0,32) | 0,22 (0,09) |
| **Tauro-conjugated bile acids** | | | | | | | |
| Taurochenodeoxycholic acid | TCDCA | 96,61 (89,70) | 15,29 (7,10) | 67,79 (54,16) | 98,51 (55,11) | 28,09 (10,05) | 18,36 (10,19) |
| Taurocholic acid | TCA | 2,21 (4,12) | 1,32 (2,09) | 1,01 (1,62) | 1,1 (0,91) | 0,86 (2,33) | 0,45 (1,10) |
| Taurodeoxycholic acid | TDCA | < Limit of detection | | | | | |
| Taurohyocholic acid | THCA | 159,25 (148,68) | 23,69 (35,93) | 149,13 (126,64) | 184,66 (88,80) | 70,63 (20,17) | 45,94 (26,11) |
| Taurohyodeoxycholic acid | THDCA | 107,78 (75,80 | 13,04 (14,98 | 88,16 (66,23 | 104,68 (58,81 | 39,07 (11,03 | 20,06 (9,757 |
| Taurolithocholic acid | TLCA | < Limit of detection | | | | | |
| Tauromuricholic acid | TMCA | 4,64 (4,78) | 1,57 (3,31) | 3,66 (2,07) | 3,23 (2,59) | 2,51 (2,47) | 2,1 (1,73) |
| Tauroursodeoxycholic acid | TUDCA | 1,43 (1,82) | 0,52 (0,3) | 1,52 (0,93) | 1,2375 (0,4) | 0,86 (0,84) | 0,63 (0,51) |
| **Glyco-conjugated bile acids** | | | | | | | |
| Glycochenodeoxycholic acid | GCDCA | 190,95 (178,94) | 29,20 (58,00) | 205,10 (87,53) | 302,92 (145,73) | 67,02 (47,56) | 48,75 (32,77) |
| Glycocholic acid | GCA | 3,97 (2,21) | 1,62 (1,20) | 5,44 (6,23) | 4,04 (2,25) | 3,02 (1,88) | 1,27 (1,32) |
| Glycodeoxycholic acid | GDCA | < Limit of detection | | | | | |
| Glycohyocholic acid | GHCA | 1214,91 (907,35) | 137,19 (289,78) | 1117,35 (784,83) | 1187,51 (589,85) | 489,67 (219,55) | 278,05 (108,94) |
| Glycolithocholic acid | GLCA | < Limit of detection | | | | | |
| Glycoursodeoxycholic acid | GUDCA | 2,42 (2,16) | 0,71 (1,18) | 2,19 (3,49) | 3,26 (2,01) | 1,1 (0,61) | 0,85 (0,58) |
|  | | | | | | | |
| **Taurine (ng/mL)** |  | 85,49 (42,24) | 74,53 (27,57) | 68,80 (32,90) | 74,70 (46,77) | 65,57 (61,82) | 67,62 (69,11) |
