# Supplementary material for: The mood stabilizers lithium and valproate disrupt hepatic and intestinal farnesoid X receptor signalling and increase bile synthesis in the rat
Source: Exp Physiol. 2025 Mar 28;110(9):1233–53. doi: 10.1113/EP092451 (PMC12400835; doi:10.1113/EP092451)
Supplement: Supplementary file 4 — Table S5. List of SYBR Green probes used in the study. [file EPH-110-1233-s005.docx]

**Table S5. List of SYBR Green probes used in the study**

| **Gene** | **Common gene name** | **Sequence (5’ → 3’)**  **left** | **Sequence (5’ → 3’)**  **right** | **Company** |
| --- | --- | --- | --- | --- |
| ***Acta2*** | Alpha-actin-2 | CCAGTCGCCATCAGGAAC | TGTGCTGTCTTCCTCTTCACA | Eurofins Genomics |
| ***Actb*** | Actin beta | CCCGCGAGTACAACCTTCT | CGTCATCCATGGCGAACT | Eurofins Genomics |
| ***Asbt***  ***Bsep***  ***Cdkn1a***  ***Cyp7a1***  ***Cyp7b1***  ***Cyp27a1***  ***Fgf19***  ***Fxr***  ***Hmgcr***  ***Ilbp***  ***Il-1β***  ***Il-6*** | Apical sodium-dependent bile acid transporter  Bile salt export pump  Apical sodium-dependent bile acid transporter  Cytochrome P450 family 7 subfamily A member 1  Cytochrome P450 family 7 subfamily B member 1  Cytochrome P450 family 27 subfamily A member 1  Fibroblast growth factor 19  Farnesoid X receptor  3-hydroxy-3-methylglutaryl-CoA reductase  Ileal lipid-binding protein  Interleukin 1 beta  Interleukin 6 | AACGATTGTGATCCCCTACG  GGGCAGTCACACCCATCTAC  GACATCTCAGGGCCGAAA  GTGAAGTCCTCCTTAGCTGTG  CACCTTGCTGGTCCCAGT  TTCCAGCTATTTCTACGAGGCTAT  TGTAGCCCAAACAGTCCATT  CCACGACCAAGCTATGCAG  GACCTTTCTAGAGCGAGTGCAT  CTCTTGCTTACACGCTCGTAG  TGTGATGAAAGACGGCACAC  CCCTTCAGGAACAGCTATGAA | CAATGGAAACAGGAATAACAAGC  CTTTATCGAGGAGTGAAAAAGTCC  GGCGCTTGGAGTGATAGAAA  CAAGTGCAACTGAATGACCTG  CAAGGGAGGTTCACAAGGAC  CCGTACTTGGCCTTGTTCA  GTTGCTCTGAAGACAATTGCC  TCTCTGTTTGCTGTATGAGTCCA  CGCTATATTCTCCCTTACTTCATCC  CCCAACTATCACCAGACTTCG  CTTCTTCTTTGGGTATTGTTTGG  ACAACATCAGTCCCAAGAAGG | Eurofins Genomics  Eurofins Genomics  Eurofins Genomics  Integrated DNA Technologies  Eurofins Genomics  Eurofins Genomics  Integrated DNA Technologies  Integrated DNA Technologies  Eurofins Genomics  Integrated DNA Technologies  Eurofins Genomics  Eurofins Genomics |
| ***Il-10***  ***Mrp2***  ***Mrp3***  ***Mrp4***  ***Myd88***  ***Ntcp***  ***Ost-α***  ***Ost-β***  ***Shp***  ***Sod2***  ***Tlr4***  ***Tnf-α*** | Interleukin 10  Multidrug resistance-associated protein 2  Multidrug resistance-associated protein 3  Multidrug resistance-associated protein 4  Myeloid differentiation primary response 88  Na^+^/taurocholate cotransporting polypeptide  Organic solute transporter alpha subunit  Organic solute transporter beta subunit  Small heterodimer partner  Superoxide dismutase 2  Toll-like receptor 4  Tumor necrosis factor alpha | AGTGGAGCAGGTGAAGAATGA  TCTCCTCCCAAATACCTCTCC  GTGCTGGCAGGCAAGACT  GCCAGACCTGGTGAGTTGTT  CCGTGAGGATATACTGTATGAACTG  AAGGGGGACATGAACCTCA  CAATTTCCACTGAGCCCAAT  GCTGCTTCTTTCGATTTCTGTT  TCCAGGACTTCACACAATGC  GGCCATATCAATCACAGCATT  GGATGATGCCTCTCTTGCAT  TGAACTTCGGGGTGATCG | TCATGGCCTTGTAGACACCTT  CTCCATCACCCTCTTCAATATCC  AAGCACAATGATAAAGTCCGTCT  CGCTCAACAGGGACGACT  TTTCTGCTGGTTGCGTATGT  CATCATGCCCAAGGCACT  CCAGGTACACAGCAGATCTTC  ATGCTTTGGTATTTCCGTTCAG  GTCCCAAGGAGTACGCATAC  TAGCCTCCAGCAACTCTCCT  TGATCCATGCATTGGTAGGTAA  GGGCTTGTCACTCGAGTTTT | Eurofins Genomics  Integrated DNA Technologies  Eurofins Genomics  Eurofins Genomics  Eurofins Genomics  Eurofins Genomics  Integrated DNA Technologies  Integrated DNA Technologies  Integrated DNA Technologies  Eurofins Genomics  Eurofins Genomics  Eurofins Genomics |
